# Enhancing quality and yield of recombinant secretory IgA antibodies in *Nicotiana benthamiana* by endoplasmic reticulum engineering

**DOI:** 10.1111/pbi.14576

**Published:** 2025-01-16

**Authors:** Kathrin Göritzer, Stanislav Melnik, Jennifer Schwestka, Elsa Arcalis, Margit Drapal, Paul Fraser, Julian K‐C. Ma, Eva Stoger

**Affiliations:** ^1^ Institute for Infection and Immunity St George's University of London London UK; ^2^ Institute of Plant Biotechnology and Cell Biology, Department of Applied Genetics and Cell Biology University of Natural Resources and Life Sciences Vienna Austria; ^3^ Royal Holloway University of London Egham UK

**Keywords:** secretory IgA, plant molecular farming, endomembrane system, recombinant protein production, monoclonal antibody, host cell engineering

## Abstract

The production of complex multimeric secretory immunoglobulins (SIgA) in *Nicotiana benthamiana* leaves is challenging, with significant reductions in complete protein assembly and consequently yield, being the most important difficulties. Expanding the physical dimensions of the ER to mimic professional antibody‐secreting cells can help to increase yields and promote protein folding and assembly. Here, we expanded the ER in *N. benthamiana* leaves by targeting the enzyme CTP:phosphocholine cytidylyltransferase (CCT), which catalyses the rate‐limiting step in the synthesis of the key membrane component phosphatidylcholine (PC). We used CRISPR/Cas to perform site‐directed mutagenesis of each of the three endogenous CCT genes in *N. benthamiana* by introducing frame‐shifting indels to remove the auto‐inhibitory C‐terminal domains. We generated stable homozygous lines of *N. benthamiana* containing different combinations of the edited genes, including plants where all three isofunctional CCT homologues were modified. Changes in ER morphology in the mutant plants were confirmed by *in vivo* confocal imaging and substantially increased the yields of two fully assembled SIgAs by prolonging the ER residence time and boosting chaperone accumulation. Through a combination of ER engineering with chaperone overexpression, we increased the yields of fully assembled SIgA by an order of magnitude, reaching almost 1 g/kg fresh leaf weight. This strategy removes a major roadblock to producing SIgA and will likely facilitate the production of other complex multimeric biopharmaceutical proteins in plants.

## Introduction

*Nicotiana benthamiana* is a plant widely used for the production of recombinant proteins (Oh *et al*., 2021; Qiu *et al*., 2014; Ward *et al*., 2021; Zahmanova *et al*., 2023). It is particularly suitable for the production of monoclonal antibodies (mAbs) based on monomeric immunoglobulins (e.g. IgG and IgA), which are assembled efficiently and produced at high yields (Virdi and Depicker, 2013). Complex antibodies such as secretory IgA (SIgA) are more challenging. This is true in all heterologous expression systems, and even in mammalian cells, they require additional technological solutions, such as reassociation *in vitro* (Bhaskara *et al*., 2021; Boullier *et al*., 2009; Puligedda *et al*., 2020). Monomeric IgA consists of two identical heavy and light chains, forming two Fab arms for antigen binding and an Fc region associated with effector functions, but it can also assemble intracellularly with a joining chain (JC) to form dimers (dIgA). At mucosal sites, dIgA associates extracellularly with a secretory component (SC) that mediates transcytosis into mucosal secretions and confers stability in the mucosal environment (Bohländer, 2023; Kilian and Russell, 2015). In plants, the whole SIgA complex is assembled in the secretory pathway, which plays a critical role in protein synthesis, folding and assembly, post‐translational modification and quality control. This includes the dimerization of mIgA by J chain and the further association of dIgA with SC (Ma *et al*., 1995).

Expanding the physical dimensions of the endoplasmic reticulum (ER), the first part of the secretory pathway (Kriechbaumer and Brandizzi, 2020), is a conserved mechanism in eukaryotes to promote protein production and to alleviate ER stress (Bommiasamy *et al*., 2009; Schuck *et al*., 2009). It can be induced by the unfolded protein response (UPR), a cellular mechanism to counteract negative consequences of augmented protein production. The expansion reduces the likelihood of protein aggregation and enhances the cell's secretory capacity (Schuck *et al*., 2009). This strategy is observed in mammalian plasma cells, which secrete antibodies equivalent to their own mass every day and manage the load by expanding the ER volume threefold during their maturation from B cells to fully differentiated plasma cells (Wiest *et al*., 1990).

**Figure 1:**
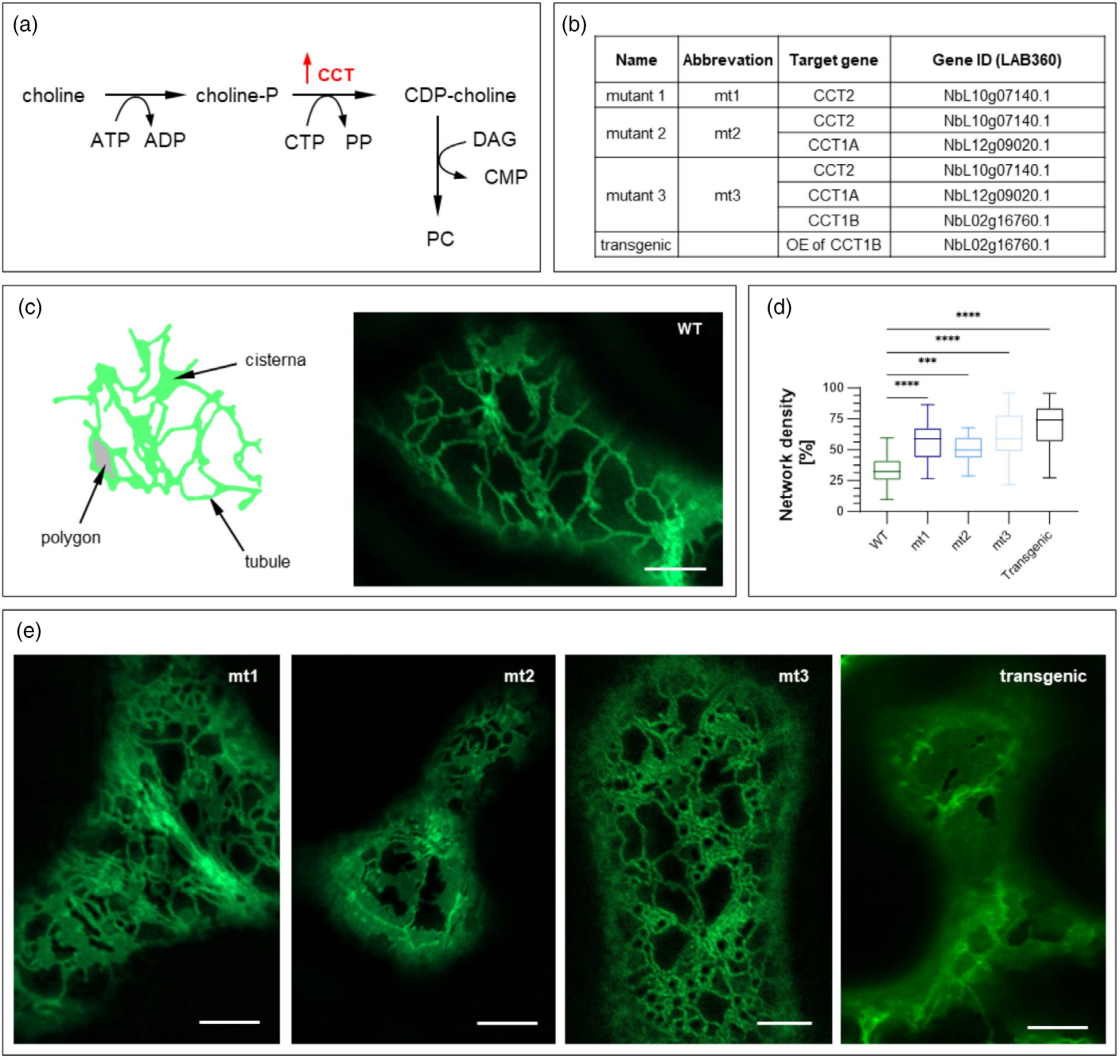
Expansion of the ER induced by genetic engineering to deregulate lipid synthesis in *Nicotiana benthamiana*. (a) Schematic and simplified representation of phosphatidyl choline (PC) synthesis. Red arrow indicates the upregulation of CTP:phosphocholine cytidyltransferase (CCT) by deleting the auto‐inhibitory C‐terminal domain. (b) Overview of gene‐edited lines. Transgenic plant carries a truncated *CCT1B* gene under the control of the enhanced CaMV 35S promoter. (c) Schematic drawing and visualization of endoplasmic reticulum (ER) morphology based on the mNeonGreen‐KDEL marker protein for confocal microscopy, depicting the tubular ER structures in wild‐type (WT) plants. (d) Network density (%) of genetically engineered *N. benthamiana* plant lines: mt1, mt2, mt3, and the transgenic line overexpressing one of the modified *CCT* genes. The density was calculated using the Vessel Analysis Plugin in ImageJ. Mean values represent 10 images from three biological replicates. Statistical significance was determined using an unpaired *t*‐test (ns, not significant, *P ≤ 0.05, **P ≤ 0.01, ***P ≤ 0.001). (e) Visualization of ER morphology using the mNeonGreen‐KDEL marker, showing the high abundance of ER sheets in mt1–3 and the transgenic line. Scale bar: 5 μm.

Phospholipids are key constituents of eukaryotic cell membranes. Their quantity and composition determine the structure and functionality of membranes. Among them, phosphatidyl choline (PC) is the major glycerolipid of most membranes (Ohlrogge and Browse, 1995). The rate‐limiting enzyme for PC synthesis is CTP:phosphocholine cytidylyltransferase (CCT, EC 2.7.7.15), which is subject to feedback regulation at its lipid‐sensing C‐terminal domain (Cornell and Ridgway, 2015). The latter has an autoinhibitory function and acts as a metabolic sensor in plant cells, monitoring membrane lipid composition and adjusting the rate of PC biosynthesis in the cell accordingly. Upon binding to anionic lipids such as phosphatidic acid (PA), the enzyme turns from a soluble inactive to a membrane‐bound active form and converts phosphocholine into CDP‐choline, a precursor for PC (Figure 1a). Since the ER membrane is mainly comprised of PC, an increase in PC concentration is generally associated with ER proliferation, as previously shown in yeast and mammalian cells (Schuck *et al*., 2009). We hypothesized that employing a gene‐editing approach to remove the autoinhibitory C‐terminal domain and constitutively activate CCT in *N. benthamiana* leaves would trigger ectopic ER expansion, thus enhancing SIgA production and assembly. An approach based on a similar principle in yeast led to ER expansion followed by enhanced IgG antibody assembly and secretion in *Saccharomyces cerevisiae* (de Ruijter *et al*., 2016).

Here, we show that the ER in *N. benthamiana* leaves can be expanded by targeting the biosynthesis of phosphatidylcholine. Using CRISPR/Cas, mutations were introduced between the catalytic and the lipid sensing domain of three endogenous CCT genes, such that the resulting proteins were truncated and rendered constitutively active. The edited plants showed an overexpanded ER morphology, which promoted the production and assembly of two SIgA antibodies. The antibodies showed an extended ER retention time in the mutant plants, and a higher accumulation of binding immunoglobulin protein (BiP) was induced upon antibody expression. The overexpression of molecular chaperones in the ER has also been shown to favour the accumulation of recombinant proteins, and we used this approach to complement our ER‐expansion strategy. For example, it was shown in previous studies that the overexpression of selected molecular chaperones in the plant ER can facilitate increased accumulation of recombinant proteins including dimeric IgA (Göritzer *et al*., 2020; Margolin *et al*., 2020a, 2020b). The combined approach of ectopic ER expansion and chaperone expression increased SIgA yields even further and has the potential to enhance the production of a broad range of complex, multimeric recombinant proteins in plants.

## Results

### *CCT* gene editing expands the ER in *N. benthamiana*

We identified three *N. benthamiana* CCT genes based on multiple alignments of the coding sequences and putative catalytic domains: two homoeologs of *CCT1* and one *CCT2* (NbSC LAB360 gene IDs NbL12g09020.1, NbL02g16760.1 and NbL10g07140.1). The CCT1A, CCT1B and CCT2 coding sequences each comprised eight exons interspersed with seven introns, and featured N‐terminal catalytic and auto‐inhibitory C‐terminal lipid‐sensing domains. To confirm the function of the C‐terminal domains, truncated versions of CCT1A, CCT1B and CCT2 comprising the first 211, 212 and 201 amino acids, respectively, were transiently expressed along with KDEL‐tagged mNeonGreen by agroinfiltration (Figure S1A). Confocal imaging revealed an expanded ER with more abundant sheets for all three CCT proteins, compared to the tubular network in wild‐type (WT) controls (Figure S1B). To exclude an additional or disturbing effect due to the marker protein mNeonGreen, we used an additional ER marker (moxGFP) and observed the same morphological phenotype (Figure S1C) (Costantini *et al*., 2015). Lipid measurements confirmed that the PC content had increased by 60% (Figure S1D).

Next, we used CRISPR/Cas9 to edit the three endogenous *CCT* genes with gRNA target sites causing a frameshift in exon 6, thus truncating the proteins before the lipid‐sensing domain while retaining catalytic activity (Table S1). The resulting plants were screened by PCR with exon‐specific primers for each *CCT* gene (Table S2) followed by Sanger sequencing, and three genome‐edited lines with different combinations of the appropriately modified *CCT* genes were selected for further analysis (Figure 1b). The edited genomic DNA sequences surrounding the target sites and the corresponding amino acid sequences are summarized in Figure S2. Most of the mutations were single‐nucleotide insertions (although we also recovered an 8‐bp deletion in *CCT2*), resulting in a frameshift and premature stop codon between the catalytic and lipid‐sensing domains. We selected three lines, one with homozygous edits in *CCT2* (mt1), one with homozygous edits in *CCT2* and *CCT1A* (mt2), and a third with edits in all three genes (mt3). The latter could not be recovered in a homozygous state, suggesting that at least one copy with the regulatory lipid‐sensing domain may be required for plant development. We also recovered a stable transgenic line overexpressing a truncated version of *CCT1B* under the control of the constitutive CaMV 35S promoter. Morphological changes in the ER were evaluated using the KDEL‐tagged mNeonGreen marker as above, showing an increase in ER volume in all four lines compared to WT controls, with the network density almost doubling. This was mainly due to more abundant ER sheets (Figure 1c–e). The similar ER densities of mt1–3 indicated that the mutation of multiple *CCT* genes does not cause additive effects. Overexpression of the truncated *CCT1B* gene under a strong viral promoter increased the proportion of ER sheets even further, but this was associated with severely impaired growth and a lower biomass compared to the edited lines, so the transgenic approach was abandoned.

### ER‐engineered plants produce and assemble SIgA with higher efficiency

**Figure 2:**
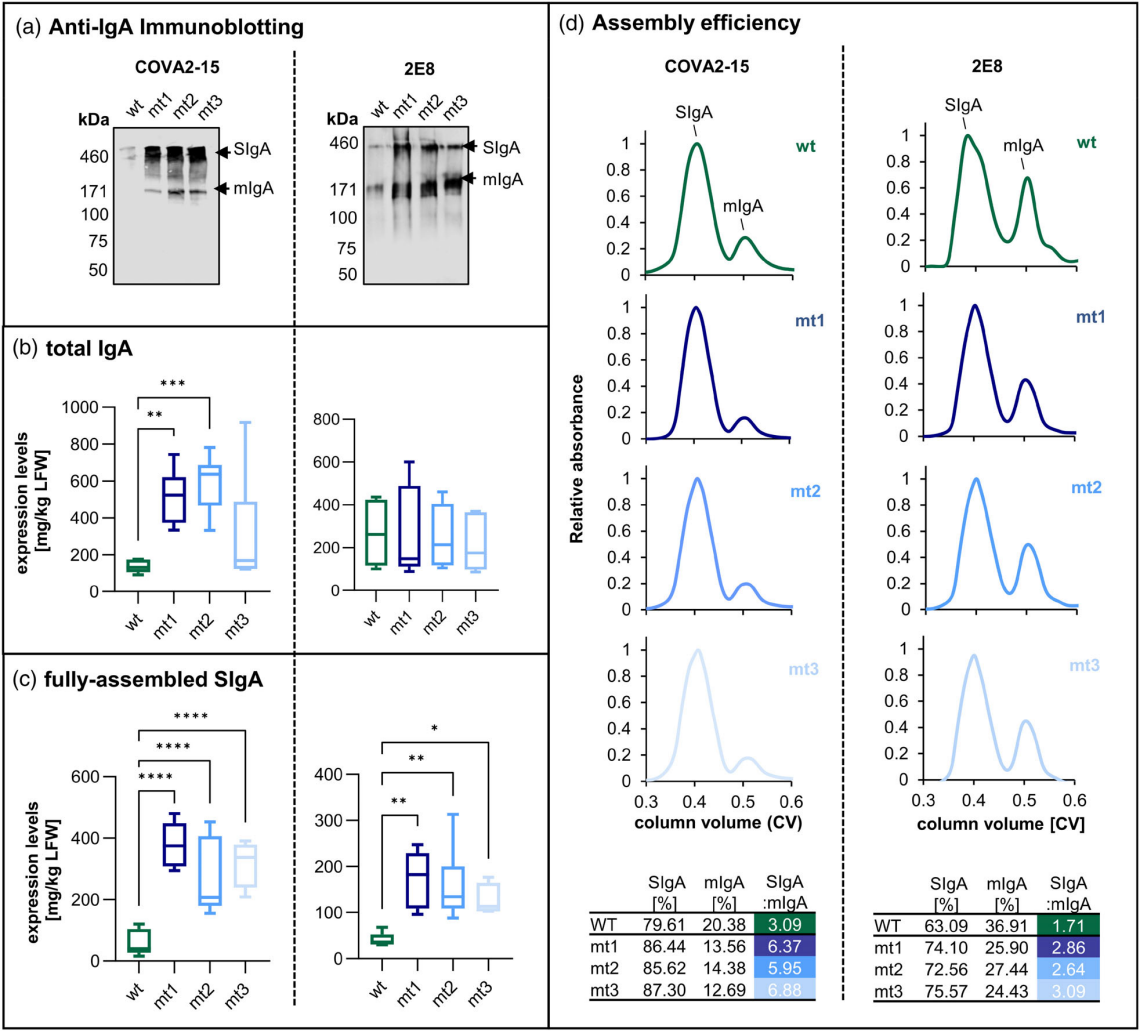
Expression and assembly of transiently expressed COVA2‐15 and 2E8 SIgA1. (a) Immunoblots of crude leaf extract from WT and ER‐engineered lines (mt1–3) with IgA heavy‐chain detected using antisera under non‐reducing conditions. (b) Total IgA1 content and (c) amount of fully‐assembled SIgA1 COVA2‐15 and 2E8 antibodies in WT and ER‐engineered lines (mt1–3). Mean values represent two technical replicates and three biological replicates for each line. Statistical significance was determined by one‐way ANOVA comparing WT to mt1, mt2 and mt3 groups (ns, not significant, *P ≤ 0.05, **P < 0.01, ***P < 0.001; ****P < 0.0001). (d) Representative size‐exclusion chromatograms of affinity‐purified antibody from one of two independent purifications. Ratio of area under the peaks is depicted in table. The major peak with the shorter retention time corresponds to the polymeric structure of fully‐assembled SIgA.

We transiently expressed two neutralizing SIgA1 antibodies against SARS‐CoV‐2, COVA2‐15 and 2E8 (Göritzer *et al*., 2024), in the three mutant lines and WT controls. After 5 days, immunoblotting showed higher IgA levels in all three ER‐engineered lines compared to WT controls (Figure 2a). Furthermore, enzyme‐linked immunosorbent assays (ELISAs) indicated higher levels of functional IgA1 and fully assembled SIgA1. Specifically, COVA2‐15 IgA1 was 3.7‐fold, 4.3‐fold and 2.5‐fold more abundant in lines mt1, mt2 and mt3, respectively, compared to WT (Figure 2b, Table S4) and the assembled multimeric SIgA1 was 6.7‐fold, 5‐fold and 5.5‐fold more abundant in the same lines, based on the absolute value of 56.7 mg/kg in WT plants (Figure 2c, Table S4). The average levels of 2E8 IgA1 were not significantly elevated in the mutant lines (Figure 2b) but the fully assembled SIgA1 was fourfold, fourfold and threefold more abundant in lines mt1, mt2 and mt3, respectively, based on the absolute value of 40.5 mg/kg in WT plants (Figure 2c, Table S4).

Affinity purification and size‐exclusion chromatography (SEC) were used to compare the relative amounts of fully assembled SIgA1 and intermediates. The major peak in all chromatograms (~400 kDa) represented the fully assembled SIgA1 (Figure 2d) and the minor peak with a higher retention time (170 kDa) represented monomeric IgA (Göritzer *et al*., 2024). Area integration of the two peaks showed that 79.6% of the COVA2‐15 SIgA1 was assembled in the WT plants, increasing to 86.4% in the ER‐engineered lines. The corresponding values for 2E8 SIgA1 were 63.1% in WT plants increasing to 74.1% in the ER‐engineered lines.

To confirm that CCT truncation rather than any off‐target effects of gene editing were responsible for the enhanced SIgA production, we transiently co‐expressed the antibody components in WT plants along with the truncated CCT1A, CCT1B and CCT2 constructs that increase ER‐network density (Figure S1). Consistent with our earlier findings, this improved the yield of assembled SIgA1, with truncated CCT2 showing the greatest effect (Figure S3). However, the overall effect was less pronounced than in stable *CCT* mutant lines (Figure 2), where mt1 (mutation in *CCT2*) also demonstrated the most consistent performance. We therefore selected mt1 for subsequent experiments.

### SIgA1 assembles in the ER and is retained for longer in the expanded ER of mt1 plants

**Figure 3:**
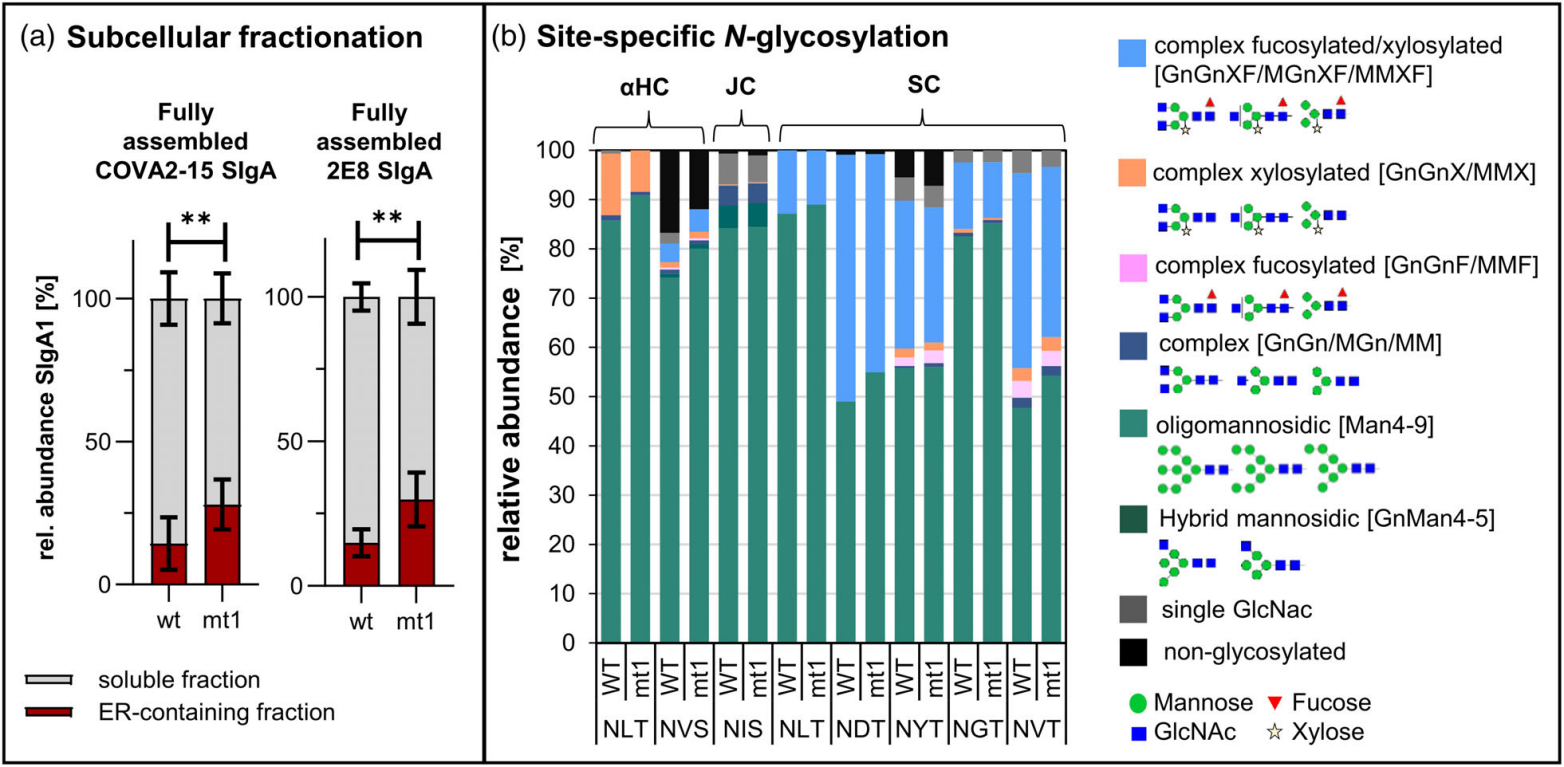
Subcellular distribution of SIgA1 in *N. benthamiana* WT and mt1 cells. (a) Relative abundance of SIgA1 in the soluble fractions and ER‐containing fractions of leaf extracts applied to a density gradient as determined by ELISA. Mean values represent two technical replicates of six biological replicates for each line. Statistical significance was determined using an unpaired *t*‐test (ns, not significant, *P ≤ 0.05, **P ≤ 0.01, ***P ≤ 0.001). (b) Site‐specific *N*‐glycosylation of purified mAbs. Bars represent the relative abundance (%) of glycoforms at each site on the heavy chain (αHC IgA1; NLT), joining chain (JC; NIS) and secretory component (SC; NLT+ NGT, NDT, NYT, NGT, NVT). *N*‐glycans are abbreviated according to the ProGlycAn system (www.proglycan.com). The symbols for the monosaccharides are drawn according to the nomenclature from the Consortium for Functional Glycomics.

To gain insight into the assembly and secretion dynamics of SIgA1 in mt1 and WT plants, we used linear sucrose gradients to separate soluble and organelle‐containing fractions of different densities from leaves expressing COVA2‐15, 2E8 or an empty vector control, followed by an RBD antigen‐binding sandwich ELISA. The fully‐assembled SIgA antibodies were predominantly found in the low‐density top fractions, typically containing soluble vacuolar and apoplastic proteins (Frigerio *et al*., 2001). A smaller amount of fully‐assembled SIgA was detected in ER‐containing (microsomal) fractions with densities of 1.16–1.22 g/mL, identified by the presence of BiP (Figure S4). This is consistent with SIgA assembly taking place in the ER, and with a post‐Golgi compartment or the intercellular space being the main accumulation site. The analysis of pooled low‐density and microsomal fractions for total IgA and fully assembled SIgA1 showed a relative abundance of fully assembled SIgA1 in the soluble fractions of 85.7 ± 3.7% (COVA2‐15 SIgA in WT), 72.0 ± 3.6% (COVA2‐15 SIgA in mt1), 85.1 ± 1.9% (2E8 SIgA1 in WT), and 70.1 ± 3.8% (2E8 SIgA1 in mt1) (Figure 3a). The secretion of COVA2‐15 and 2E8 SIgA1 therefore appears slower in the ER‐engineered plants given the higher relative abundance of fully assembled SIgA1 in the ER‐containing fractions of the mt1 line compared to WT controls.

SIgA1 is heavily glycosylated, with two *N*‐glycosylation sites found in the heavy chain, one in the JC and six in the SC. *N*‐linked glycans are attached in the ER and processed in the Golgi apparatus, so glyco‐analysis can give insight into the processing, secretion and localization of recombinant proteins. To assess the glycosylation status of COVA2‐15 and 2E8 SIgA1 produced in mt1 and WT plants, the purified antibodies were digested with trypsin followed by LC‐ESI‐MS analysis (Figure 3b, Table S5). As expected, each *N*‐glycosylation site had a unique glycan composition, probably reflecting differences in accessibility for glycan‐modifying enzymes. For example, one site in the IgA heavy chain (NLT) and the single site in the JC mostly featured oligomannosidic glycans (Man_5–9_) and only small quantities of complex bi‐antennary glycans. This probably reflects the presence of the SC, which is attached in the ER and hinders access to the *N*‐glycosylation sites in the CH2 domain of the heavy chain and the JC (Göritzer *et al*., 2024). We observed site‐specific *N*‐glycan processing at all six positions on the SC, producing varying proportions of complex bi‐antennary (MMXF, GnMXF, GnGnXF and GnGnF) and oligomannosidic (Man_4–9_) glycans. The exposed sites (NDT, NYT, NGT and NVT) featured more fully processed complex *N*‐glycans. Importantly, a consistent shift towards a higher proportion of oligomannosidic *N*‐glycans (Man_4–9_) was found at all sites on COVA2‐15 and 2E8 SIgA1 in line mt1. This aligns well with the earlier observation that a greater proportion of fully assembled COVA2‐15 and 2E8 SIgA1 is found in the ER of line mt1 compared to WT controls.

Among the fully processed complex glycans attached to the SC, there was a remarkably high proportion of paucimannosidic structures (MMXF and GnMXF), which are often found in the vacuole (Shin *et al*., 2017). We therefore isolated protoplasts and subsequently vacuoles from leaves infiltrated with COVA2‐15, and confirmed that a portion of SIgA1 remains within the cell, and at least partially accumulates in the vacuole (Figure S5).

### Chaperone accumulation is modified in ER‐engineered plants during the UPR

**Figure 4:**
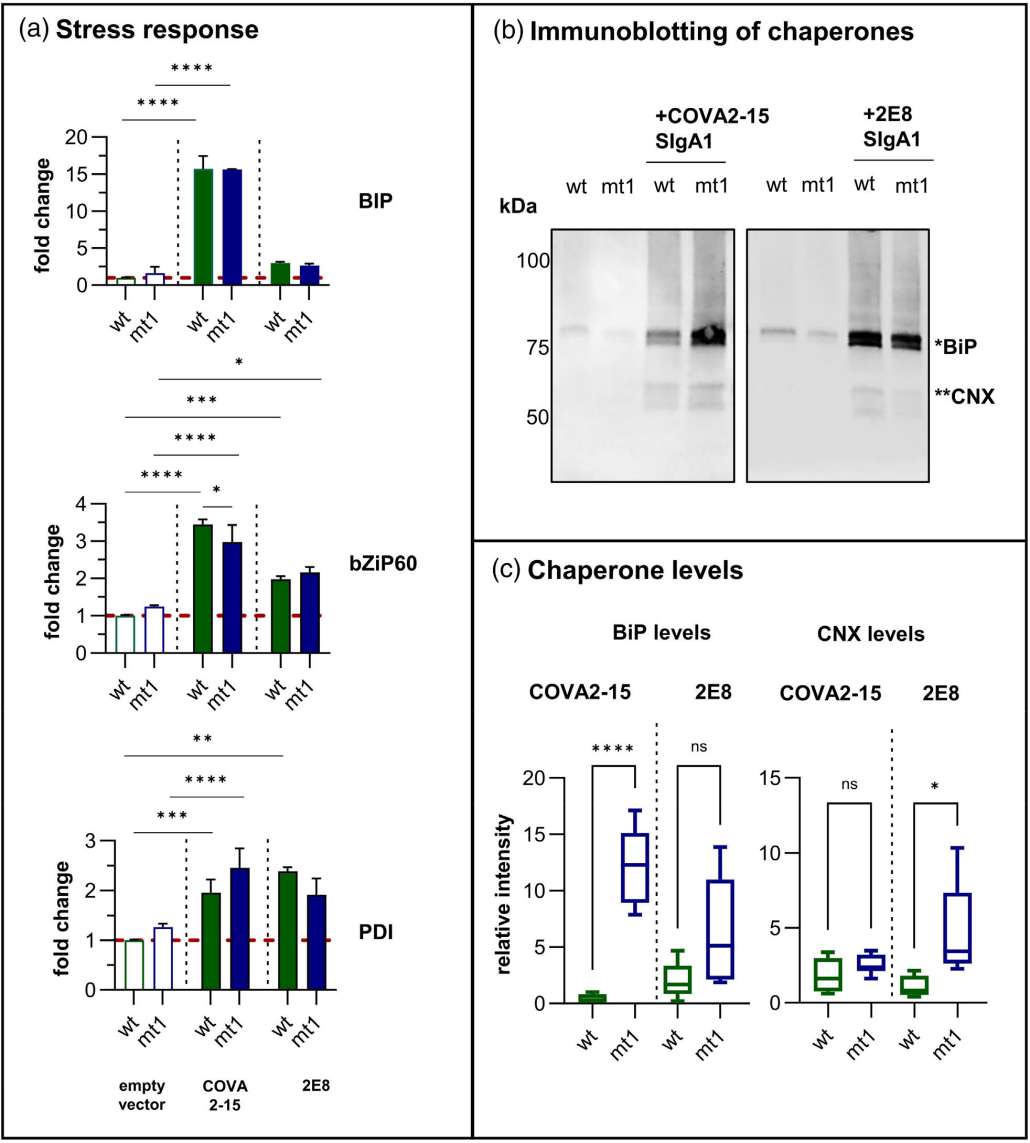
ER stress‐related genes and chaperone expression levels. (a) qRT‐PCR analysis of ER stress‐related genes. The relative transcript levels of *BiP*, *bZIP60* and *PDI* are shown following the infiltration with an empty vector control (pEAQ‐HT, left) or with COVA2‐15 and 2E8 SIgA1. Reference genes: 18S rRNA and L25. Mean values represent three technical replicates of three pooled biological replicates for each line. (b) Immunoblotting of WT and mt1 extracts infiltrated with empty vector control or SIgA and detected with anti‐BiP and anti‐CNX antisera. (c) Densitometry, showing the relative intensity of BiP and calnexin protein levels in WT and mt1 plants. Mean values represent the relative intensity of six biological replicates. Statistical significance was determined using an unpaired *t*‐test (ns, not significant, *P ≤ 0.05, **P ≤ 0.01, ***P ≤ 0.001, ****P < 0.0001).

The effect of ER‐engineered lines on the ER stress response (reflected by chaperone induction) was determined by first comparing the basal transcription levels of ER stress markers such as *BiP*, *bZIP60* and *PDI* (Hamorsky *et al*., 2015; Margolin *et al*., 2020a, 2020b) in the empty vector control and plants expressing SIgA. There was no significant difference between WT and mt1 plants expressing the empty vector control (Figure 4a), indicating that *CCT* editing and ER expansion did not trigger the UPR. However, both WT and mt1 plants showed a significant increase in ER stress markers when COVA2‐15 SIgA1 was expressed, with *BiP* upregulated by 15‐fold and *bZiP* and *PDI* upregulated by two‐ to fourfold. Similar results were observed for 2E8 SIgA1 although with less pronounced upregulation of the stress‐related genes. Overall, the stress response at the transcriptional level did not differ significantly between mt1 and WT plants. However, immunoblot analysis revealed a significantly higher BiP level in mt1 plants expressing COVA2‐15 SIgA1 compared to WT plants expressing the same SIgA1 (Figure 4b,c). BiP levels also tended to be higher in mt1 plants compared to WT when both expressed 2E8 SIgA1, but the difference was not statistically significant. Calnexin, a glycoprotein‐specific chaperone, was slightly more abundant in mt1 plants than WT controls both expressing 2E8 SIgA1, whereas the difference between mt1 and WT plants both expressing COVA2‐15 SIgA1 was not significant. These results show that *CCT* editing did not induce a constitutive UPR or alter the transcriptional signature of the UPR during SIgA expression. However, BiP in particular accumulated to higher levels in mt1 than WT plants following UPR induction.

### Heterologous overexpression of ER‐resident chaperones boosts SIgA yields even further

**Figure 5:**
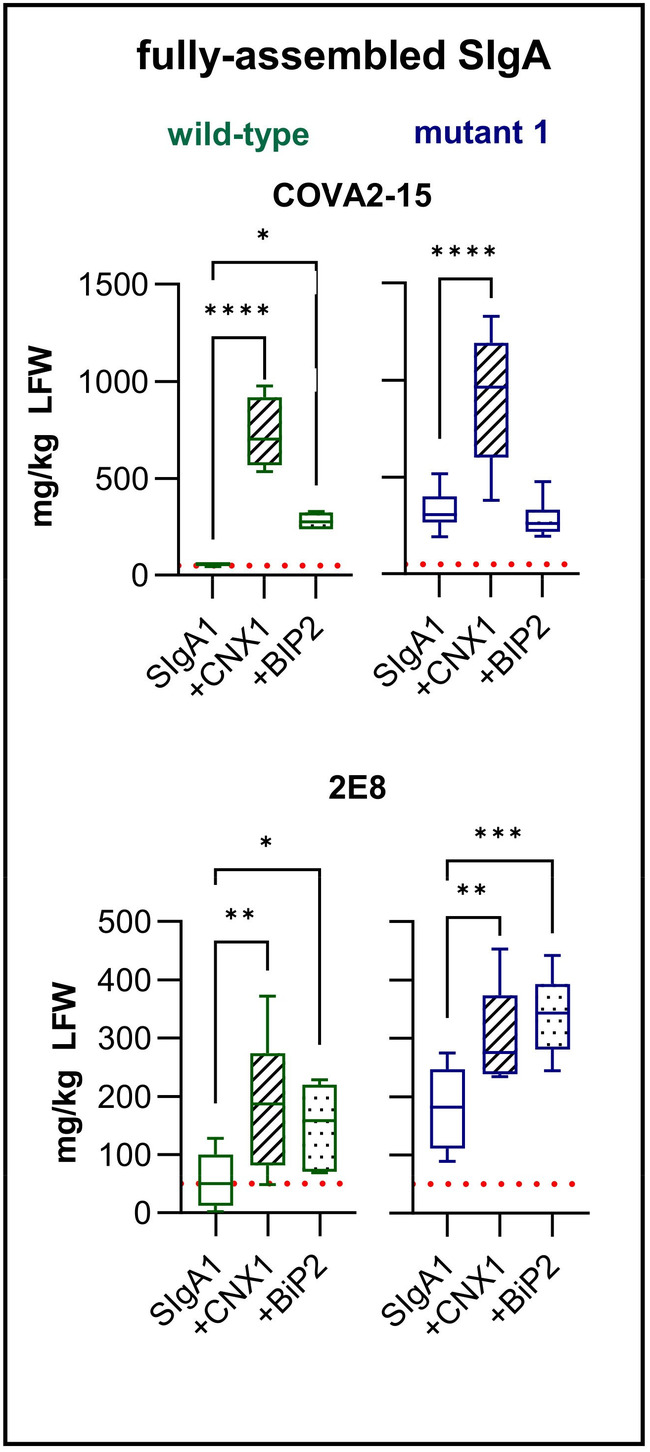
Overexpression of heterologous chaperones can further boost SIgA expression in WT and mutant plants. Fully‐assembled COVA2‐15 and 2E8 SIgA1 in WT and mt1 lines were quantified when co‐expressed alone or in combination with BiP2 and CNX1. Mean values represent two technical replicates of three biological replicates for each line. Statistical significance was determined by one‐way ANOVA comparing WT to mt1 (ns, not significant, *P ≤ 0.05, **P < 0.01, ***P < 0.001, ****P < 0.0001).

Finally, we evaluated the transient co‐expression of exogenous chaperones with SIgA in the ER because this strategy was previously shown to increase the accumulation of recombinant proteins including dIgA (Göritzer *et al*., 2020; Margolin *et al*., 2020a). We co‐expressed COVA2‐15 and 2E8 SIgA1 with the *Arabidopsis thaliana* chaperones BiP2 and CNX1 (Figure 5). Leaves were harvested 5 days post infiltration and the fully assembled and functional SIgA1 was quantified as before (Figure 5). The individual expression of either BiP2 or CNX1 was able to increase the yields of COVA2‐15 and 2E8 SIgA1, although there were differences between the two antibodies. In WT plants, BiP2 expression increased the yield of COVA2‐15 SIgA1 by 5‐fold to 280 mg/kg, whereas CNX1 expression increased the yield by 13‐fold to 730 mg/kg. Similar results were achieved for 2E8 SIgA1, but the increase in yield was only 3–4‐fold, reaching 150–190 mg/kg. In the mt1 line, BiP2 expression did not increase the yields of COVA2‐15 SIgA1 any further, whereas CNX1 caused a further threefold increase to 910 mg/kg. The combined strategy of *CCT* editing and CNX1 overexpression therefore achieved a 16‐fold increase in the yield of fully‐functional and assembled COVA2‐15 SIgA1 compared to expression without chaperones in WT plants. For 2E8, both BiP2 and CNX1 resulted in a yield of approximately 300 mg/kg, representing a 7‐fold increase compared to WT plants without heterologous chaperones.

## Discussion

Professional secretory cells, such as antibody‐secreting plasma cells, have an expanded ER morphology composed mainly of ribosome‐decorated sheets, which forms as a normal part of cellular differentiation preceding the upregulation of immunoglobulin gene transcription (Tellier and Nutt, 2019; van Anken *et al*., 2003). Similarly, the plant endomembrane system in seeds has abundant ER sheets to support the synthesis of storage proteins (Arcalís *et al*., 2020). Conversely, the ER architecture is far less prominent in leaves, which are often used for recombinant protein production and consequently have to cope with high protein loads.

We therefore modified the ER of *N. benthamiana* leaves to explore the impact of preemptive ER expansion. This process requires additional PC building blocks, so we targeted CCT (catalysing the rate‐limiting step of PC synthesis) by removing the autoinhibitory C‐terminal lipid‐binding domain (Cornell and Ridgway, 2015) and thereby triggering ER proliferation. This is consistent with earlier studies involving the overexpression of truncated Arabidopsis *CCT1* (Craddock *et al*., 2015). We found that the strategy works equally well with *N. benthamiana CCT1* and *CCT2*, suggesting they are functionally redundant, like their orthologs in Arabidopsis (Inatsugi *et al*., 2009). The transient expression of a truncated *CCT* was sufficient to induce ER proliferation, but its effect on SIgA antibody production was moderate compared to that observed in stably edited plants, possibly due to the limited time available for completing the structural transition of the ER. Stable overexpression of truncated CCT on the other hand yielded only a single viable line that was unsuitable for antibody production due to severe dwarfing and low biomass. In contrast, the editing of endogenous *CCT* genes produced stable mutants with excellent performance and normal growth. The modification of a single *CCT* gene was sufficient to achieve the desired ER morphological alteration, with no additive effects gained by stacking mutations in multiple *CCT* genes. This observation potentially suggests the existence of overriding regulatory mechanisms within the plants. The importance of maintaining at least one *CCT* copy with an intact lipid‐binding domain was demonstrated by the inability to generate complete homozygous knockout lines in *N. benthamiana* with all *CCT* genes truncated. This finding parallels the observation that deletion of the *Pcyt1a* gene encoding the CCT1 ortholog CCTα in mammals results in embryonic lethality (Wang *et al*., 2005).

The expanded ER in *CCT* mutants had a positive effect on SIgA production and quality of assembly, possibly because ER expansion increases the luminal volume, allowing the accommodation of nascent polypeptides and potentially reducing misfolding and aggregation events (Schuck *et al*., 2009). Additionally, subcellular fractionation and *N*‐glycan analysis revealed that SIgA was retained for longer in the expanded ER, which may provide additional time for the antibody components to engage with each other and with chaperones. Similarly, SIgA assembly was previously shown to be promoted by expressing KDEL‐tagged SC and heavy chain components causing ER retention (Juarez *et al*., 2013). Furthermore, the expanded ER also provides more room for chaperones, which are needed for proper protein folding and assembly. However, the enlarged ER alone does not trigger the UPR or the upregulation of chaperones, as evidenced by the basal levels of ER stress markers we observed. Chaperone expression levels were similarly unaffected in ER‐modified yeast cells lacking a lipid‐regulator gene (Schuck *et al*., 2009).

However, the UPR was triggered by the expression of SIgA components, and although the transcriptional signature of this UPR was similar in WT and ER‐engineered plants expressing COVA2‐15, the actual amount of BiP was much higher in the edited lines. The higher accumulation of BiP might be possible through the increased ER volume providing a more diluted environment for BiP. Endogenous calnexin levels only showed a marginal increase in the CCT‐mutant compared to WT plants. Conversely, the heterologous expression of calnexin further boosted COVA2‐15 expression levels in the enlarged ER environment of the mutant plants, whereas heterologous BiP2 overexpression did not, likely due to the already elevated levels of endogenous BiP.

Chaperones such as BiP, calnexin and PDI are often co‐expressed to enhance recombinant protein production, but the efficiency of this strategy is product‐dependent (Kim *et al*., 2003; Klabunde *et al*., 2007; Smith *et al*., 2004). For example, a high copy number of BiP genes reduced the secretion of recombinant β‐glucosidase in yeast, whereas PDI overexpression had the opposite effect, possibly reflecting a product‐dependent UPR (Smith *et al*., 2004). The general positive effect of chaperones can be attributed to their ability to bind target proteins, prolonging their residence time in the ER, which promotes proper folding and assembly (Muresan and Arvan, 1997). The longer retention time of SIgA1 in the ER‐engineered plants could therefore also reflect the greater amount of BiP in the enlarged ER environment. After exiting the ER, SIgA was partially transported to the vacuole, consistent with the presence of paucimannosidic structures probably generated by β‐hexosaminidases primarily located in the vacuole (Shin *et al*., 2017). This finding agrees with previous reports showing that IgAs produced in plants are inefficiently secreted due to the presence of a sorting motif for vacuolar localization (Hadlington *et al*., 2003; Paul *et al*., 2014; Westerhof *et al*., 2014).

Our study suggests that expanding the physical dimensions of the plant ER and optimizing its chaperone profiles could improve the production of complex recombinant biotherapeutics, including SIgAs (Margolin *et al*., 2020b; Zhou *et al*., 2018). Although fully‐functional SIgAs have been produced in plants before, the limited yields and inefficient assembly have hindered their development as mucosal biologics (Ma *et al*., 1995; Paul *et al*., 2014; Virdi and Depicker, 2013). After removing this bottleneck, topical passive delivery of plant‐made neutralizing SIgA1 antibodies to mucosal surfaces could be an important adjunct to systemic vaccination and a valuable strategy for the treatment of infectious deceases. SIgA antibodies have higher stability and neutralization capacity in mucosal tissues compared to IgG antibodies, and also interact with mucus to enhance its protective potential through mechanisms such as mucous trapping (Göritzer *et al*., 2024; Ma *et al*., 1995; Paul *et al*., 2014; Teh *et al*., 2020).

Our study demonstrates that refining plant ER morphology, inspired by the sophisticated secretory pathways of professional secretory mammalian systems, is successful and can eventually go beyond mere physical expansion. We have shown that chaperone optimization and physical expansion of the ER can increase the yield of two fully assembled SIgA in plants by up to 16‐fold suggesting this is a general effect that would be applicable to other complex recombinant proteins.

## Experimental procedures

### Plant material and growth conditions

*Nicotiana benthamiana* (LAB strain) WT, *CCT* mutant and transgenic plants were grown under long‐day conditions (16‐h photoperiod). Sterile seedlings and tissue cultures were maintained in an incubator at 25 °C under long‐day conditions. After rooting, T0 primary transformants were transferred to soil and grown in a growth chamber at 24 °C under long‐day conditions. All subsequent generations were propagated directly in the growth chamber starting from seeds.

### Phylogenetic analysis

We identified the *N. benthamiana* genes encoding CCT (EC 2.7.7.15) by locating all putative *CCT* sequences in the *N. benthamiana* genome databases using BLAST based on published records. We screened the *N. benthamiana* Sequencing Consortium (NbSC) database (https://www.nbenth.com/), the Queensland University of Technology database (https://benthgenome.qut.edu.au/) and the Sol Genomics Network (https://solgenomics.net/). The genomic DNA, gene and protein sequences were aligned using the MAFFT algorithm L‐INS‐I (Katoh and Standley, 2013).

The positions of the catalytic domain in the N‐terminal part of the protein and the lipid‐interacting amphipathic helix in the C‐terminal part were inferred by multiple sequence alignment based on sequence similarity to homologous proteins from other species where 3D structures and experimental data were available, such as those from *N. tabacum* (catalytic domain boundaries based on AlphaFold models AF‐A0A1S3XUJ7‐F1, AF‐A0A1S4CAW1‐F1, AF‐A0A1S3YTV1‐F1), *A. thaliana* (AlphaFold models AF‐Q9ZV56‐F1, AF‐F4JJE0‐F1), *Saccharomyces cerevisiae* (AF‐P13259‐F1), and *Rattus norvegicus* (data on lipid‐interacting amphipathic motif, and X‐ray models 3HL4, 4MVC, 4MVD) (Johnson and Cornell, 1994).

### Design of gRNAs

We used CCTop software (https://cctop.cos.uni‐heidelberg.de:8043/) to identify putative target sites with Tobacco ‘*Nicotiana benthamiana* Niben101’ set as the reference genome. The parameters were set to 20 nt maximum length and 12 nt seed sequence length allowing up to four mismatches for the identification of off‐targets and up to two mismatches in the core region. We selected gRNAs with no predicted off‐targets within other genes and that targeted the region between the catalytic and lipid‐sensing domains of each *CCT* gene (Table S1).

### Construct design for gene editing

For CRISPR/Cas9 genome editing, we used *Streptococcus pyogenes* Cas9 (SpCas9) and a PolII‐transcribed multiplex gRNA system in which concatenated transcripts of gRNAs are processed by Csy4 ribonuclease (Čermák *et al*., 2017). The gene‐editing vector pDV003 was constructed by transferring a pre‐assembled polycistronic sgRNA cassette from pMGC03 to a Golden Gate destination vector pDVM1 using AarI. The cassette consisted of the CmYLCV promoter followed by two sgRNAs (with G5 and G4.2 spacers) featuring an optimized scaffold (Dang *et al.*, 2015). Each guide was flanked by Csy4 recognition sites. The vector pDVM1 is a derivative of pDIRECT_21C (Čermák *et al*., 2017) with an added *ccdB* gene flanked by AarI sites in place of the template sequence used to amplify sgRNA cassette elements and a FMV34S promoter‐driven transcription unit for plastid‐targeted mCherry FP. The sgRNA cassette was assembled as previously described (Čermák *et al*., 2017) except an intermediate plasmid pUV3 rather than the final vector was first used for the assembly. Two separate plasmids (pYLCV1 and pOGS1) were used as PCR templates to generate one promoter‐containing and two sgRNA scaffold‐containing elements. Primers employed for the amplification of cassette elements are listed in Table S2.

### Stable transformation of *N. benthamiana*

*CCT* mutant lines were prepared as previously described (Uetz *et al*., 2022). Briefly, *N. benthamiana* seeds were germinated on Murashige and Skoog (MS) medium (Duchefa, the Netherlands), and after 6 days the seedlings were inoculated with *Agrobacterium tumefaciens* GV3101(pMP90) carrying pDV003 or expression constructs (pTE291, pTE292 or pTE30) encoding truncated versions of CCT. After inoculation, the cotyledons were incubated for 3 days in the dark at 25 °C before transfer to selection medium containing 100 mg/L kanamycin and 100 mg/L timentin. Regenerating shoots were rooted and transferred to soil for screening.

### Mutant screening

Genomic DNA from stably transformed T0 plants was isolated from leaves and the T0 plants were first screened by PCR using primers 35S_F and Dm1_Cas9_304_R to confirm the presence of the *SpCas9* transgene (Uetz *et al*., 2022). Cas9‐positive plants were then screened using target‐site‐specific primers (Table S2), followed by Sanger sequencing to identify frame‐shifting mutations. Homozygous, *SpCas9‐*free mutants were identified in the next generation as previously described (Uetz *et al*., 2022).

### Target protein constructs design and cloning

Cloning procedures for the heavy and light chain genes of the two human anti‐SARS‐CoV‐2 IgA mAbs (COVA2‐15 and 2E8), and the human SC and JC constructs, as well as procedures for the transformation of *A. tumefaciens* GV3101 (Leibniz Institut DSMZ‐Deutsche Sammlung von Mikroorganismen und Zellkulturen GmbH, DSM 12364) have been described elsewhere (Göritzer *et al*., 2024). Truncated *N. benthamiana* CCT1A, CCT1B and CCT2 sequences encoding the first 211, 212 and 201 amino acids, respectively, were inserted into a plant expression vector derived from pTRA between the CaMVC 35S promoter and terminator (Figure S1A). The mNeonGreen construct (pTRAkc‐mNG‐K) was generated by inserting the synthetic mNeonGreen‐KDEL sequence (manually optimized for dicots and ordered from GeneCust) into the same plant expression vector. The moxGFP construct was kindly provided by A. Goossens (Costantini *et al*., 2015). Constructs for the overexpression of ER‐resident chaperones BiP2, CNX1 and CRT2 from *A. thaliana* are described elsewhere (Göritzer *et al*., 2020).

### Transient expression in *N. benthamiana*

Cultures of *A. tumefaciens* GV3101 were grown overnight shaking at 28 °C in lysogeny broth (LB) containing 25 μg/mL rifampicin and 50 μg/mL kanamycin and were diluted in infiltration medium (10 mm MES pH 5.6, 10 mm MgSO_4_, 100 μm acetosyringone). Heavy and light chain constructs of SIgA1 were diluted to an OD_600_ of 0.05 and mixed with the JC construct at an OD_600_ of 0.2 and the SC construct at an OD_600_ of 0.1. We then infiltrated the leaves of 6‐week‐old gene‐edited and WT *N. benthamiana* plants which were maintained as described above for 5 days before harvesting.

### Lipid analysis

Lyophilized leaves were ground to a fine powder with a TissueRuptor (Qiagen, Venlo, the Netherlands). An aliquot of the leaf powder (10 mg) was extracted with methanol and deionized water (800 μL, 1:1, v/v) for 30 min and another 30 min after the addition of chloroform (800 μL). The whole extraction was performed at 40 rpm with a rotator SB3 (Stuart/Col‐eParmer St. Neots, Cambridgeshire, UK) at 4 °C. Samples were then centrifuged for 5 min at 4 °C and 20 000 ***g***. The chloroform phase of each sample (1 μL injection volume) was analysed with an Atalantis HILIC Silica column (100 × 2.1 mm, 3 μm, Waters, Milford, MA, USA) and an Infinity II1290 UHPLC coupled to a 6550 iFunnel QTof (Agilent Technologies, Inc., Santa Clara, CA, USA) as previously described (Drapal *et al*., 2022). PC content was quantified with a dose–response curve constructed from an authentic standard (Sigma, Gillingham, Dorset, UK).

### ELISA

We coated Nunc MaxiSorp 96‐well plates (Thermo Fisher Scientific) overnight at 4 °C with 100 ng/well of RBD‐His and blocked with phosphate‐buffered saline (PBS, pH 7.4) containing 0.1% (v/v) Tween‐20 (PBST) and 5% bovine serum albumin (Sigma‐Aldrich/Millipore, Milwaukee, WI, USA)) as previously described (Göritzer *et al*., 2024). Crude leaf extracts were prepared by disruption in a bead mill with five volumes of ice‐cold PBST. Samples were centrifuged twice for 10 min at 20 000 ***g*** to obtain clarified extracts. These were applied to the ELISA plates in serial dilutions and incubated for 2 h at 37 °C. Then the plates were incubated with either horseradish peroxidase (HRP)‐conjugated goat polyclonal antiserum against human IgA (abcam ab97215, diluted 1:5000 in blocking solution) or a mouse mAb against human SC (Sigma‐Aldrich I6635, diluted 1:2000 in blocking solution) followed by an HRP‐conjugated anti‐mouse IgG (Sigma‐Aldrich, A9917, diluted 1:5000 in blocking solution). Finally, we added TMB substrate (Thermo Fisher Scientific, Waltham, MA, USA), stopped the reaction with 2 m H_2_SO_4_ and read the absorbance at 450 nm on an Infinite F200 Pro plate reader (Tecan, Männedorf, Switzerland).

### Affinity purification and size‐exclusion chromatography (SEC)

Snap‐frozen plant material was ground with a precooled mortar and pestle and mixed with three volumes of PBST extraction buffer (containing 40 mm ascorbic acid, pH 6.8). Homogenized leaf material was passed through a Miracloth filter (Merck Millipore, Burlington, MA, USA), centrifuged at 20 000 ***g*** for 1 h, and passed through a Durapore 0.45‐μm membrane filter (Merck Millipore). Clarified extracts were purified using Pierce Protein A resin (Thermo Fisher Scientific) for COVA2‐15 IgA, or CaptureSelect IgA affinity matrix (Thermo Fisher Scientific) for 2E8 IgA. This was possible because COVA2‐15 IgA contains a heavy chain from the human VH3 gene family, which contains a binding site for Protein A in the variable domain (Ghose *et al*., 2005). The resins were packed in columns and pre‐equilibrated with PBS (pH 7.4). Bound antibodies were eluted using 0.1 m glycine (pH 2.7) with immediate neutralization in 10% (v/v) 1 m Tris–HCl (pH 8.0). Fractions containing the antibody were pooled and dialyzed against PBS overnight at 4 °C using a Slide‐A‐Lyzer cassette with a 10‐kDa molecular weight cut‐off (Thermo Fisher Scientific). Pooled and dialyzed protein fractions were concentrated using Amicon centrifugal filters with a molecular weight cut‐off of 100 kDa (Merck Millipore). We then injected 100 μg of affinity‐purified antibody onto a HiLoad 16/600 Superdex 200 pg SEC column (GE Healthcare, Chicago, IL, USA) equilibrated with PBS (pH 7.4) connected to an ÄKTA pure FPLC system (GE Healthcare). Peak integration to determine the ratios of polymeric and monomeric IgA variants was carried out with the Unicorn Analysis Software v7.0.

### Confocal laser scanning microscopy (CLSM)

Before recombinant protein expression, leaf sections from three biological replicates of each edited plant line (plus WT controls) were analysed by CLSM using a Leica SP5 microscope. ER morphology was visualized using the ER‐retained marker protein mNeonGreen‐KDEL (excitation 506 nm, emission 517 nm). Ten images of each replicate were captured with the same laser parameters and pixel size of ≤85 nm. Images produced by Leica LAS software were processed using LASX (Leica Microsystems UK) and ImageJ (NIH). The network density was calculated as the ER area/total area × 100 using the Vessel Analysis Plugin in ImageJ (https://imagej.net/plugins/vessel‐analysis).

### Extraction of apoplastic fluid

Leaves were harvested 5 days post infiltration (dpi) and intracellular fluid was collected by low‐speed centrifugation as previously described (Castilho *et al*., 2011).

### Subcellular fractionation on isopycnic sucrose gradients

For subcellular fractionation (Arcalís *et al*., 2022), transfected plant material was ground in a pre‐cooled mortar and pestle and resuspended in four volumes of extraction buffer (16% (w/w) sucrose in 100 mm Tris–HCl pH 7.8, 10 mm KCl, 2 mm MgCl_2_) and 600 μL of the extract was loaded onto a precooled linear sucrose gradient (12–55% (w/w) sucrose in 100 mm Tris–HCl pH 7.8, 10 mm KCl, 2 mm MgCl_2_) and centrifuged at 155 000 ***g*** at 4 °C for 2 h. We recovered 16 fractions, each 650 μL, from the top. Six biological replicates were loaded onto an individual sucrose cushion for individual centrifugation runs. Fractions containing soluble vacuolar and apoplastic proteins (fractions with a density ρ of 1.065–1.105 g/mL) and ER‐containing fractions (fractions with a density ρ of 1.155–1.235 g/mL and confirmed to contain BiP) were pooled separately for sandwich ELISA to determine the total IgA and SIgA content. Relative abundance refers to the measured amount of antibody in a pooled fraction relative to the total amount of sample in the entire gradient.

### Protoplast preparation and vacuole isolation

Vacuoles were isolated from leaf mesophyll protoplasts as previously described (Robert *et al*., 2007) with modifications. Briefly, young fully expanded *N. benthamiana* leaves were infiltrated with enzyme solution (1% Cellulase Onozuka RS, 1% Macerozime R‐10 and 250 mg/L timentin in 0.4 m sucrose, 125 mm CaCl_2_ and 10 mm MES, pH 5.6) and placed in a Petri dish with the abaxial side in contact with enzyme solution overnight at RT. Protoplasts were recovered from the solution by centrifuging for 20 min at 60 ***g*** using a swing‐bucket rotor. Vital protoplasts were collected from the floating green band, washed twice with wash buffer (0.4 m mannitol, 10 mm MES, pH 5.6) and observed by light microscopy. Pre‐warmed lysis buffer was added to the protoplasts followed by ultracentrifugation on a Ficoll gradient at 71 000 ***g*** and 10 °C. Vacuoles were collected from the interface (Robert *et al*., 2007).

### RT‐qPCR analysis of ER stress‐related genes

Total RNA was extracted from 100 mg of snap‐frozen leaf tissue harvested 5 dpi. The material was homogenized in a bead mill and RNA was recovered using the RNeasy Plant Mini Kit (Qiagen) according to the manufacturer's protocol. Reverse transcription and real time RT‐qPCR were carried out using the Luna Universal One‐Step RT‐qPCR Kit according to manufacturer's protocol. RT‐qPCR was used to evaluate the expression of *BiP*, *PDI* and *bZIP60* as previously described (Hamorsky *et al*., 2015).

### SDS‐PAGE and immunoblots for SIgA and chaperones

Samples were extracted under non‐reducing conditions in 4× NuPAGE LDS Sample Buffer and resolved on NuPage 4–12% Bis/Tris gels before blotting onto a nitrocellulose membrane using the Novex Semi‐Dry Blotter (Thermo Fisher Scientific). SIgA was detected using the same antibodies described above for ELISA. Chaperones were detected using rabbit polyclonal anti‐BiP (Agrisera, Vännäs, Sweden, AS09‐481, diluted 1:2000) and rabbit polyclonal anti‐calnexin (Agrisera, AS12‐2365, diluted 1:2500). Signals were detected using the Amersham ECL Prime Western Blotting Detection Reagent (Cytiva, Marlborough, MA, USA). ChemiDoc MP (BioRad, Hercules, CA, USA) and ImageLab v6.0.1 were used for image analysis and protein quantitation.

### Glycoanalysis of SIgA by mass spectrometry

A 20‐μg sample of purified protein was reduced, S‐alkylated and digested with trypsin (Promega, Madison, WI, USA). Glycopeptides were analysed by capillary reversed‐phase chromatography and electrospray mass spectrometry using an Agilent Series 6560 LC‐IMS‐QTOFMS instrument as previously described (Göritzer *et al*., 2017).

## Conflict of interest

The authors declare no conflict of interest.

## Author contributions

Conceptualization and study design: ES, SM, JKCM, KG, JS. Experimental work and data analysis: KG, SM, JS, EA, MD, PF. Writing original draft: SM, JS, KG, ES, JKCM. Editing and revisions: all authors.

## Supporting information


**Figure S1** (A) T‐DNA region of constructs encoding truncated versions of *N. benthamiana* CCT1A/CCT1B/CCT2 comprising the first 211/212/201 amino acids of the protein. E35SP, cauliflower mosaic virus 35S promoter with a duplicated enhancer; U, chalcone synthase 5′‐UTR; 35ST, cauliflower mosaic virus 35S terminator; SAR, tobacco Rb7 scaffold attachment region; NP, nopaline synthase promoter; NPTII, neomycin phosphotransferase II; nosT, nopaline synthase terminator; LB, RB, left and right border, respectively. (B) Visualization of ER morphology using the mNeonGreen‐KDEL marker protein for confocal microscopy. Left picture shows the tubular ER structures of wild‐type (WT) plants, and the others show the high abundance of ER sheets in *N. benthamiana* plants 5 dpi. (C) Alternative visualization of ER morphology using moxGFP (Costantini *et al*., 2015). Ten images of each replicate were captured with the same laser parameters and pixel size of ≤85 nm. Images produced by Leica LAS software were processed using LASX and ImageJ. Scale bars: 5 μm. (D) Quantification of PC content in *N. benthamiana* leaves 5 dpi with empty vector control or *CCT2* construct.
**Figure S2** (A) Genomic DNA sequences at target sites in exon 6 (highlighted in gray) of the CCT homologs in the edited lines mt1, mt2 and mt3 are compared to the WT sequences. Exons are capitalized, protospacers are shown in bold and indels are underlined. (B) Amino acid sequences of protein products deduced from the modified *CCT* genes in the edited lines mt1, mt2 and mt3. WT protein sequences are provided for comparison. The N‐terminal region encompassing the catalytic domain and corresponding to the truncated CCT variant is highlighted in gray. The mutation site is underlined and the amino acid sequence from another ORF is shown in light gray.
**Figure S3** Transient co‐expression of SIgA1 with truncated CCT1A, CCT1B or CCT2 in WT *N. benthamiana* increases the production and assembly of SIgA. Levels of total IgA (A) and fully‐assembled COVA2‐15 and 2E8 SIgA1 antibodies (B) were determined by antigen sandwich ELISA. Mean values represent three replicates of three pooled biological replicates for each line. Statistical significance was determined by one‐way ANOVA comparing WT to CCT1A, CCT1B and CCT2 groups (ns, not significant, **P < 0.01, ***P < 0.001, ****P < 0.0001).
**Figure S4** Non‐reducing SDS‐PAGE followed by immunoblotting of the density gradient fractions prepared from cell extracts of *N. benthamiana* mt1 plants expressing COVA2‐15. Chaperones were detected using antisera against BiP and IgA. One representative blot is shown from six biological replicates with similar results.
**Figure S5** Non‐reducing SDS‐PAGE followed by immunoblotting of vacuolar fractions (VI‐III). Vacuoles were isolated from *N. benthamiana* WT leaves infiltrated with monomeric or secretory COVA2‐15 IgA1. Protoplasts expressing SIgA were loaded as a control. The IgA1 was detected using an αHC antibody. We also used antibodies to detect the vacuolar marker V‐ATPase and the plasma membrane marker H+ATPase. One representative experiment is shown from two biological replicates with similar results.
**Table S1** gRNA target sites.
**Table S2** Primers used for amplification and Sanger sequencing of target gene regions in the ER‐engineered plant lines.
**Table S3** Primers used to construct the gRNA cassette.
**Table S4** Expression levels of COVA2‐15 and 2E8 IgA in WT and ER‐engineered lines.
**Table S5** Site‐specific *N*‐glycosylation of COVA2‐15 and 2E8 SIgA1 in WT *N. benthamiana* and mt1 lines.

## Data Availability

The data that supports the findings of this study are available in the supplementary material of this article.
