# Supplementary material for: Enhancing quality and yield of recombinant secretory IgA antibodies in Nicotiana benthamiana by endoplasmic reticulum engineering
Source: Plant Biotechnol J. 2025 Jan 16;23(4):1178–89. doi: 10.1111/pbi.14576 (PMC11933863; doi:10.1111/pbi.14576)
Supplement: Supplementary file 1 — Figure S1 (A) T‐DNA region of constructs encoding truncated versions of N. benthamiana CCT1A/CCT1B/CCT2 comprising the first 211/212/201 amino acids of the protein. E35SP, cauliflower mosaic virus 35S promoter with a duplicated enhancer; U, chalcone synthase 5′‐UTR; 35ST, cauliflower mosaic virus 35S terminator; SAR, tobacco Rb7 scaffold attachment region; NP, nopaline synthase promoter; NPTII, neomycin phosphotransferase II; nosT, nopaline synthase terminator; LB, RB, left and right border, respectively. (B) Visualization of ER morphology using the mNeonGreen‐KDEL marker protein for confocal microscopy. Left picture shows the tubular ER structures of wild‐type (WT) plants, and the others show the high abundance of ER sheets in N. benthamiana plants 5 dpi. (C) Alternative visualization of ER morphology using moxGFP (Costantini et al., 2015). Ten images of each replicate were captured with the same laser parameters and pixel size of ≤85 nm. Images produced by Leica LAS software were processed using LASX and ImageJ. Scale bars: 5 μm. (D) Quantification of PC content in N. benthamiana leaves 5 dpi with empty vector control or CCT2 construct. Figure S2 (A) Genomic DNA sequences at target sites in exon 6 (highlighted in gray) of the CCT homologs in the edited lines mt1, mt2 and mt3 are compared to the WT sequences. Exons are capitalized, protospacers are shown in bold and indels are underlined. (B) Amino acid sequences of protein products deduced from the modified CCT genes in the edited lines mt1, mt2 and mt3. WT protein sequences are provided for comparison. The N‐terminal region encompassing the catalytic domain and corresponding to the truncated CCT variant is highlighted in gray. The mutation site is underlined and the amino acid sequence from another ORF is shown in light gray. Figure S3 Transient co‐expression of SIgA1 with truncated CCT1A, CCT1B or CCT2 in WT N. benthamiana increases the production and assembly of SIgA. Levels of total IgA (A) and fully‐assembled [file PBI-23-1178-s001.docx]

**Supplemental Information:**

**Enhancing quality and** **yield of recombinant secretory IgA antibodies in *Nicotiana benthamiana* by endoplasmic reticulum engineering**

Kathrin Göritzer*^1,2^, Stanislav Melnik*^2^, Jennifer Schwestka^2^, Elsa Arcalis^2^, Margit Drapal^3^, Paul Fraser^3^, Julian K-C. Ma**^1^, Eva Stoger**^2^

^1^Institute for Infection and Immunity, St George’s University of London, London, UK

^2^Institute of Plant Biotechnology and Cell Biology, Department of Applied Genetics and Cell Biology, University of Natural Resources and Life Sciences, Vienna, Austria

^3^Royal Holloway University of London, Egham, UK

* Joint first authors - these authors contributed equally to the work

** Joint last authors - these authors contributed equally to the work

*Correspondence: [eva.stoeger@boku.ac.at](mailto:eva.stoeger@boku.ac.at), [jma@sgul.ac.uk](mailto:jma@sgul.ac.uk)


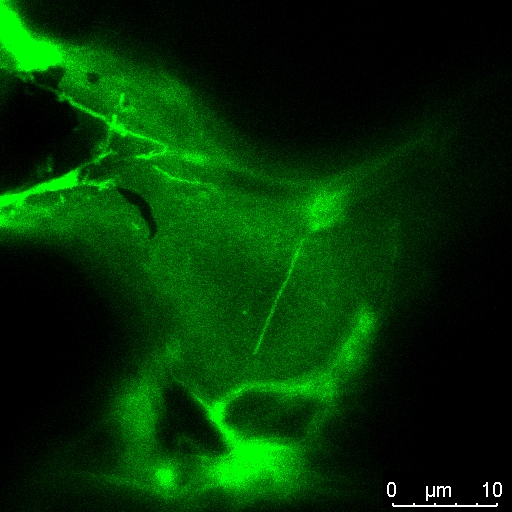


CCT1AN211

Empty vector

**B**


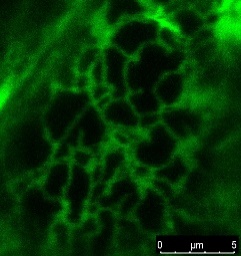

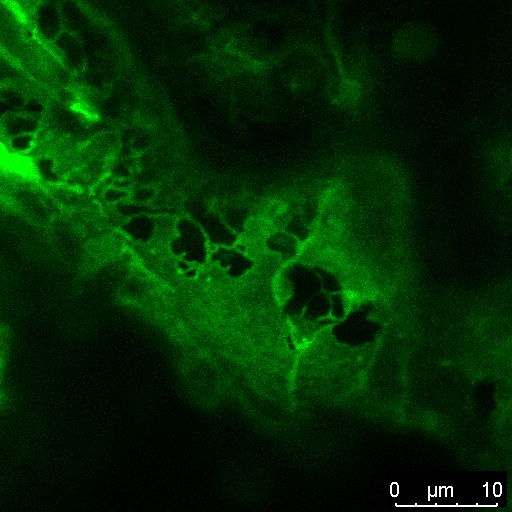

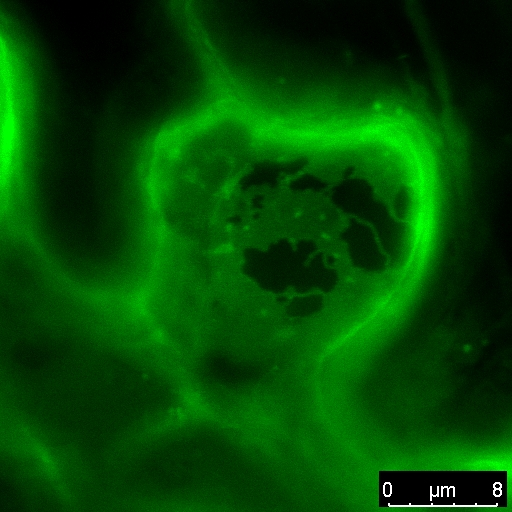


CCT1BN212

CCT2N201


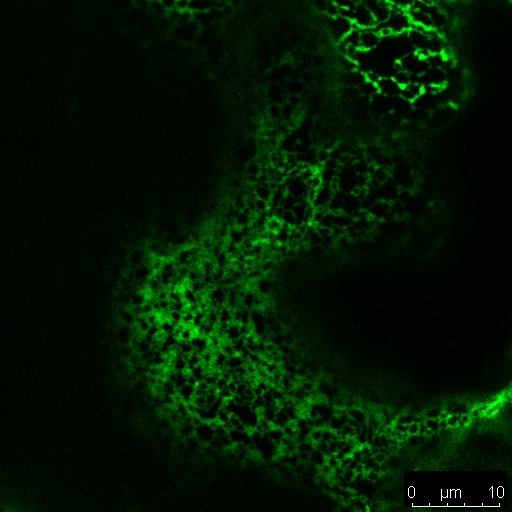

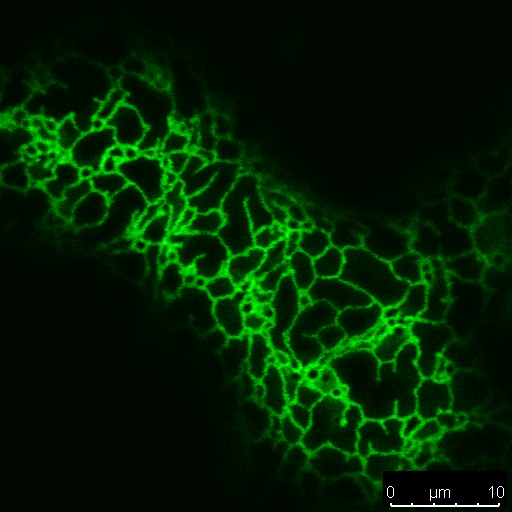


**C**

CCT2N201

Empty vector


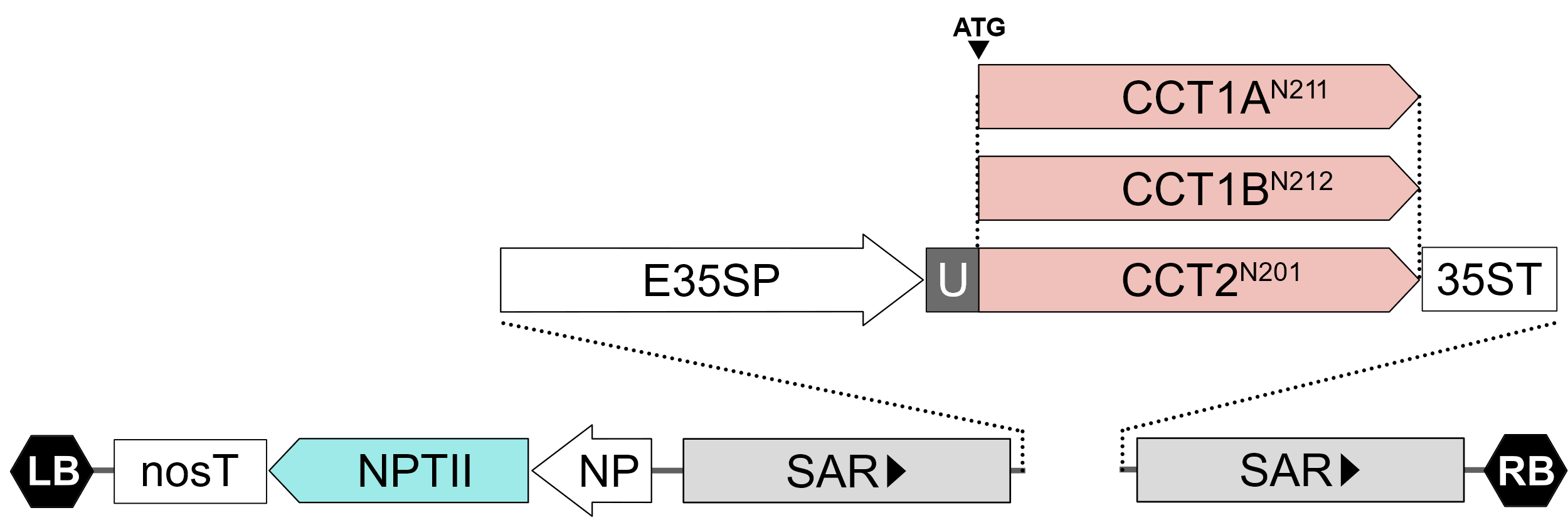


**A**

**D**

**Figure S1:** (**A**) T-DNA region of constructs encoding truncated versions of *N. benthamiana* CCT1A/CCT1B/CCT2 comprising the first 211/212/201 amino acids of the protein. E35SP – cauliflower mosaic virus 35S promoter with a duplicated enhancer; U – chalcone synthase 5′-UTR; 35ST – cauliflower mosaic virus 35S terminator; SAR – tobacco Rb7 scaffold attachment region; NP – nopaline synthase promoter; NPTII – neomycin phosphotransferase II; nosT – nopaline synthase terminator; LB, RB – left and right border, respectively. (**B**) Visualization of ER morphology using the mNeonGreen-KDEL marker protein for confocal microscopy. Left picture shows the tubular ER structures of wild-type (WT) plants, and the others show the high abundance of ER sheets in *N. benthamiana* plants 5 dpi. (**C**) Alternative visualization of ER morphology using moxGFP.(Costantini et al., 2015) Ten images of each replicate were captured with the same laser parameters and pixel size of ≤ 85 nm. Images produced by Leica LAS software were processed using LASX and ImageJ. Scale bars: 5µm. (**D**) Quantification of PC content in *N. benthamiana* leaves 5 dpi with empty vector control or *CCT2* construct.

**(A)**

**NbCCT1A gDNA target region:**

*>NbCCT1A_EX6_LAB (WT)*

ggtgatttaataccttgcctttcccagggaaaaagtgttttaattggatgatgtagactttctgctagcattagctttacatttcgctttttatttgctttgccacgtatataaactcattattgcttcttttagGAAAAAAGGCTGAGAGTGAACCGGGGGTTGAAAAAGTTGCAGGAGAGAGTAAAGAAACA**ACAAGAAAAAGTGGAAGAGA**AGgtatactcttctttatatgacttacaaactctaatgatgcatttcatttttttaatttcaaatgcaagcttgtgtttgggaataatatagttggcaattacctggaaaaaactgggggaaaggatattaaaatccctgtccttcagccgctagatcttgagttgtctgttttcaaatggcactt

*>NbCCT1A_EX6_mt2 (+1bp)*

ggtgatttaataccttgcctttcccagggaaaaagtgttttaattggatgatgtagactttctgctagcattagctttacatttcgctttttatttgctttgccacgtatataaactcattattgcttcttttagGAAAAAAGGCTGAGAGTGAACCGGGGGTTGAAAAAGTTGCAGGAGAGAGTAAAGAAACAACAAGAAAAAGTGGAAGTAGAAGgtatactcttctttatatgacttacaaactctaatgatgcatttcatttttttaatttcaaatgcaagcttgtgtttgggaataatatagttggcaattacctggaaaaaactgggggaaaggatattaaaatccctgtccttcagccgctagatcttgagttgtctgttttcaaatggcactt

*>NbCCT1A_EX6_mt3 (+1bp)*

ggtgatttaataccttgcctttcccagggaaaaagtgttttaattggatgatgtagactttctgctagcattagctttacatttcgctttttatttgctttgccacgtatataaactcattattgcttcttttagGAAAAAAGGCTGAGAGTGAACCGGGGGTTGAAAAAGTTGCAGGAGAGAGTAAAGAAACAACAAGAAAAAGTGGAAGTAGAAGgtatactcttctttatatgacttacaaactctaatgatgcatttcatttttttaatttcaaatgcaagcttgtgtttgggaataatatagttggcaattacctggaaaaaactgggggaaaggatattaaaatccctgtccttcagccgctagatcttgagttgtctgttttcaaatggcactt

**NbCCT1B gDNA target region:**

*>NbCCT1B_EX6_LAB (WT)*

CGTGAAGgtatgctctttagtgttataattgggtgatttaataccttgcctttcgcaggaaaaaagcattttaattggatgatgtagactttctgctagcattagctttacatttcactttttatttgttttgccacatattaaactcattattgcttcttttagGAAAAAAGGCTGAGAGTGAACAGGGGGTTGAAAAAGTTGCATGAGAGAGTAAAGAAACA**ACAAGAAAAAGTGGAAGAGA**AGgtatactcttctttatatgacttacaaactctaatgatgcatttcatttctttaatttcaaatgcaagcttgtggtagagcctccatagtatatgtttgtga

*>NbCCT1B_EX6_mt3 (+1bp)*

CGTGAAGgtatgctctttagtgttataattgggtgatttaataccttgcctttcgcaggaaaaaagcattttaattggatgatgtagactttctgctagcattagctttacatttcactttttatttgttttgccacatattaaactcattattgcttcttttagGAAAAAAGGCTGAGAGTGAACAGGGGGTTGAAAAAGTTGCATGAGAGAGTAAAGAAACAACAAGAAAAAGTGGAAGAAGAAGgtatactcttctttatatgacttacaaactctaatgatgcatttcatttctttaatttcaaatgcaagcttgtggtagagcctccatagtatatgtttgtga

**NbCCT2 gDNA target region:**

*>NbCCT2_EX6_LAB (WT)*

AGGAACTTGGTGTTAGCTATgtcaaggcatgatctttactcaaataaaatgttctgctttacttattttcatccttttgtttgcgtcacatggtgttcatatggtatgtgatatggccttttcatgagatattgtttcatgtatttagGAAAAGCGATTGAGGGTCAACATGAGGCTAATGAAGTTACAGGAGAAAGTCAAAGAACA**TCAAGAGAAAGTGGGAGAAA**AGgtacagtcagatgtgaaggcaatcttttgtgtgcgtttatatgatactttggttggatgtgagtaatatctggcatttttataattgatggcaagagttaagattgaattcaattcatatcttactaaatttgaaatcaaacaacttatcaattggatgatacaactacacaaatagcttaattgtcttttgcttatagctttttctttacttgattgcaacttgtcttattttatgcaagggatgatttcactttgg

*>NbCCT2_EX6_mt1 (-8bp)*

AGGAACTTGGTGTTAGCTATgtcaaggcatgatctttactcaaataaaatgttctgctttacttattttcatccttttgtttgcgtcacatggtgttcatatggtatgtgatatggccttttcatgagatattgtttcatgtatttagGAAAAGCGATTGAGGGTCAACATGAGGCTAATGAAGTTACAGGAGAAAGTCAAAGAACATCAAGAGAAAAAAGgtacagtcagatgtgaaggcaatcttttgtgtgcgtttatatgatactttggttggatgtgagtaatatctggcatttttataattgatggcaagagttaagattgaattcaattcatatcttactaaatttgaaatcaaacaacttatcaattggatgatacaactacacaaatagcttaattgtcttttgcttatagctttttctttacttgattgcaacttgtcttattttatgcaagggatgatttcactttgg

*>NbCCT2_EX6_mt2 (+1bp)*

AGGAACTTGGTGTTAGCTATgtcaaggcatgatctttactcaaataaaatgttctgctttacttattttcatccttttgtttgcgtcacatggtgttcatatggtatgtgatatggccttttcatgagatattgtttcatgtatttagGAAAAGCGATTGAGGGTCAACATGAGGCTAATGAAGTTACAGGAGAAAGTCAAAGAACATCAAGAGAAAGTGGGAGAAAAAGgtacagtcagatgtgaaggcaatcttttgtgtgcgtttatatgatactttggttggatgtgagtaatatctggcatttttataattgatggcaagagttaagattgaattcaattcatatcttactaaatttgaaatcaaacaacttatcaattggatgatacaactacacaaatagcttaattgtcttttgcttatagctttttctttacttgattgcaacttgtcttattttatgcaagggatgatttcactttgg

*>NbCCT2_EX6_mt 3 (+1bp)*

AGGAACTTGGTGTTAGCTATgtcaaggcatgatctttactcaaataaaatgttctgctttacttattttcatccttttgtttgcgtcacatggtgttcatatggtatgtgatatggccttttcatgagatattgtttcatgtatttagGAAAAGCGATTGAGGGTCAACATGAGGCTAATGAAGTTACAGGAGAAAGTCAAAGAACATCAAGAGAAAGTGGGAGAAAAAGgtacagtcagatgtgaaggcaatcttttgtgtgcgtttatatgatactttggttggatgtgagtaatatctggcatttttataattgatggcaagagttaagattgaattcaattcatatcttactaaatttgaaatcaaacaacttatcaattggatgatacaactacacaaatagcttaattgtcttttgcttatagctttttctttacttgattgcaacttgtcttattttatgcaagggatgatttcactttgg

**(B)**

**NbCCT1A protein:**

*>*NbCCT1A_LAB (WT)

MENGSEDPQLQQQQQQPLPRRLKPIPPNPPPTDRPARIYADGIYDLFHFGHARALEQAKKLLPNTYLLVGCCNDEITHMYKGKTVMNDKERYESLRHCKWVDEVIPDAPWVVTPEFIEKHQIDYVAHDALPYADASGAGNDVYEYVKSIGKFLETKRTDGISTSDLIMRIVKDYNEYVMRNLDRGYSRKDLGLSYVKEKRLRVNRGLKKLQERVKKQQEKVEEKIQTVAKHRNIWVENADRLVAGFLEMFEEGCHKMGTVIRDRIQEQIRTKSIKGLLYDKEDDDDDYDYYYGSTDDDEEYYDGEDED*

*>*NbCCT1A_mt2 and mt3 (+1bp)

MENGSEDPQLQQQQQQPLPRRLKPIPPNPPPTDRPARIYADGIYDLFHFGHARALEQAKKLLPNTYLLVGCCNDEITHMYKGKTVMNDKERYESLRHCKWVDEVIPDAPWVVTPEFIEKHQIDYVAHDALPYADASGAGNDVYEYVKSIGKFLETKRTDGISTSDLIMRIVKDYNEYVMRNLDRGYSRKDLGLSYVKEKRLRVNRGLKKLQERVKKQQEKVEVEDTNSSKASEYLGGKC*

**NbCCT1B protein:**

*>*NbCCT1B_LAB (WT)

MENGSEDPQLQLQEQQQPQPRRLKPIPPNPPPTDRPARIYADGIYDLFHFGHARALEQAKKLLPNTYLLVGCCNDEITHMYKGKTVMNDKERYESLRHCKWVDEVIPDAPWVVTPEFIDKHQIDYVAHDALPYADASGAGNDVYEYVKSIGKFLETKRTDGISTSDLIMRIVKDYNEYVMRNLDRGYSRKDLGLSYVKEKRLRVNRGLKKLHERVKKQQEKVEEKIQTVAKHRNIWVENADRLVAGFLEMFEEGCHKMGTVIRDRIQEQIRTKSIKGLLYDKEDDDDDYDYYYGSTDDDEEYYDGEDED*

*>*NbCCT1B_mt3 (+1bp)

MENGSEDPQLQLQEQQQPQPRRLKPIPPNPPPTDRPARIYADGIYDLFHFGHARALEQAKKLLPNTYLLVGCCNDEITHMYKGKTVMNDKERYESLRHCKWVDEVIPDAPWVVTPEFIDKHQIDYVAHDALPYADASGAGNDVYEYVKSIGKFLETKRTDGISTSDLIMRIVKDYNEYVMRNLDRGYSRKDLGLSYVKEKRLRVNRGLKKLHERVKKQQEKVEEEDTNSSKASEYLGGKCG*

**NbCCT2 protein:**

*>*NbCCT2_LAB (WT)

MGETGIATAEHNNQHHCANDLPPSDRPVRVYADGIYDLFHFGHARSLEQAKKSFPNTYLLVGCCSDEMTHNYKGRTVMTEEERYESLRHCKWVDEVIPDAPWVISQEFLDKHHIDYVAHDALPYADASGAGKDVYEFVKAVGRFKETKRTDGISTSDVIMRIIKDYNKYVMRNLDRGYSRKELGVSYVKEKRLRVNMRLMKLQEKVKEHQEKVGEKIQTVAKTAGMHHNEWIENADRWVAGFLQMFEESCHKMGTAIRDRIQERARGQKSRDLLDNGNTIDTDEEEDDEYYYVEEEEEEYFDNEEQHIEDKKNSSLSTK**

*>*NbCCT2_mt1 (-8bp)

MGETGIATAEHNNQHHCANDLPPSDRPVRVYADGIYDLFHFGHARSLEQAKKSFPNTYLLVGCCSDEMTHNYKGRTVMTEEERYESLRHCKWVDEVIPDAPWVISQEFLDKHHIDYVAHDALPYADASGAGKDVYEFVKAVGRFKETKRTDGISTSDVIMRIIKDYNKYVMRNLDRGYSRKELGVSYEKRLRVNMRLMKLQEKVKEHQEKKDSNCCKDSWYASQ*

*>*NbCCT2_mt2 (+1bp)

MGETGIATAEHNNQHHCANDLPPSDRPVRVYADGIYDLFHFGHARSLEQAKKSFPNTYLLVGCCSDEMTHNYKGRTVMTEEERYESLRHCKWVDEVIPDAPWVISQEFLDKHHIDYVAHDALPYADASGAGKDVYEFVKAVGRFKETKRTDGISTSDVIMRIIKDYNKYVMRNLDRGYSRKELGVSYEKRLRVNMRLMKLQEKVKEHQEKVGEKDSNCCKDSWYASQ*

*>*NbCCT2_mt3 (+1bp)

MGETGIATAEHNNQHHCANDLPPSDRPVRVYADGIYDLFHFGHARSLEQAKKSFPNTYLLVGCCSDEMTHNYKGRTVMTEEERYESLRHCKWVDEVIPDAPWVISQEFLDKHHIDYVAHDALPYADASGAGKDVYEFVKAVGRFKETKRTDGISTSDVIMRIIKDYNKYVMRNLDRGYSRKELGVSYEKRLRVNMRLMKLQEKVKEHQEKVGEKDSNCCKDSWYASQ*

**Figure S2:** (**A**) Genomic DNA sequences at target sites in exon 6 (highlighted in gray) of the CCT homologs in the edited lines mt1, mt2 and mt3 are compared to the WT sequences. Exons are capitalized, protospacers are shown in bold and indels are underlined. (**B**) Amino acid sequences of protein products deduced from the modified *CCT* genes in the edited lines mt1, mt2 and mt3. WT protein sequences are provided for comparison. The N-terminal region encompassing the catalytic domain and corresponding to the truncated CCT variant is highlighted in gray. The mutation site is underlined and the amino acid sequence from another ORF is shown in light gray.


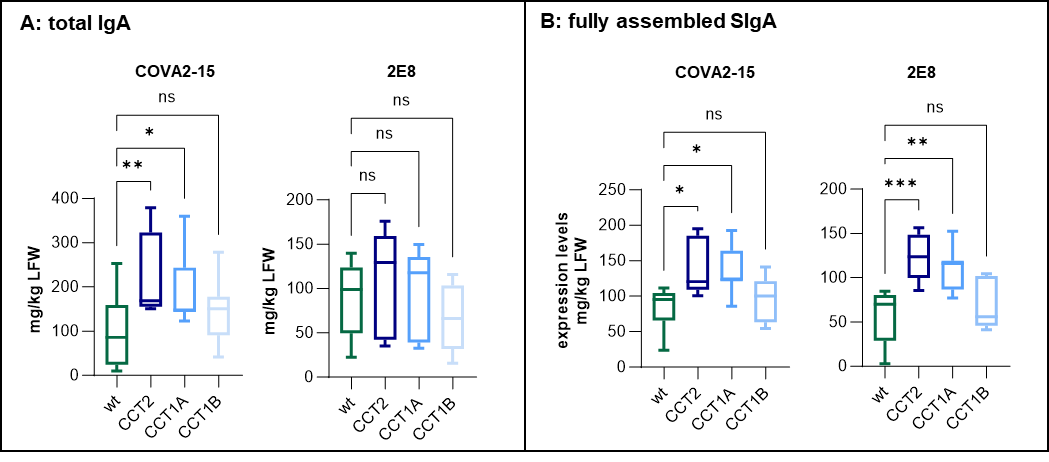


**Figure S3:** Transient co-expression of SIgA1 with truncated CCT1A, CCT1B or CCT2 in WT *N. benthamiana* increases the production and assembly of SIgA. Levels of total IgA (**A**) and fully-assembled COVA2-15 and 2E8 SIgA1 antibodies (**B**) were determined by antigen sandwich ELISA. Mean values represent three replicates of three pooled biological replicates for each line. Statistical significance was determined by one-way ANOVA comparing WT to CCT1A, CCT1B and CCT2 groups (ns, not significant, **p < 0.01, ***p < 0.001, ****p < 0.0001).

**density:**


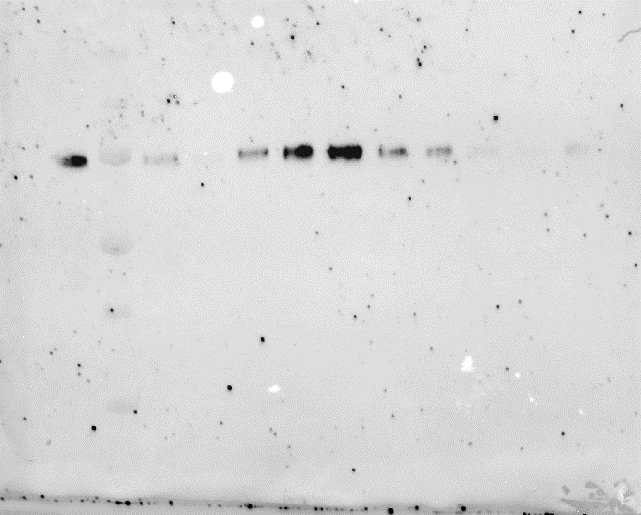


**BiP**

75 -


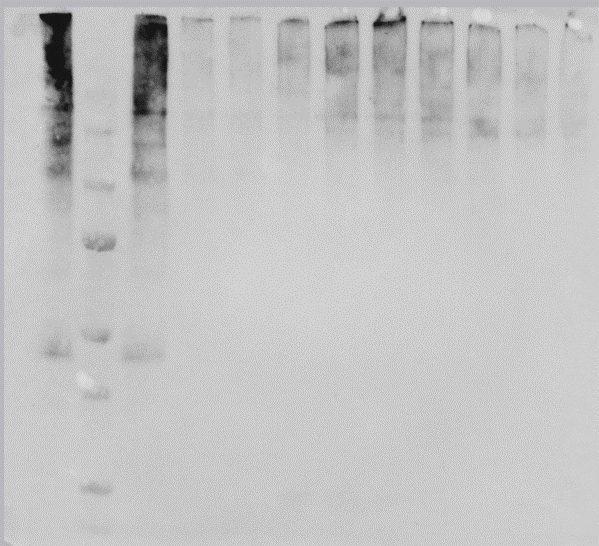


460 -

171 -

**IgA**

1.15

1.16

1.18

1.19

1.21

1.22

1.24

1.25

**kDa**

**Figure S4:** Non-reducing SDS-PAGE followed by immunoblotting of the density gradient fractions prepared from cell extracts of *N. benthamiana* mt1 plants expressing COVA2-15. Chaperones were detected using antisera against BiP and IgA. One representative blot is shown from six biological replicates with similar results.


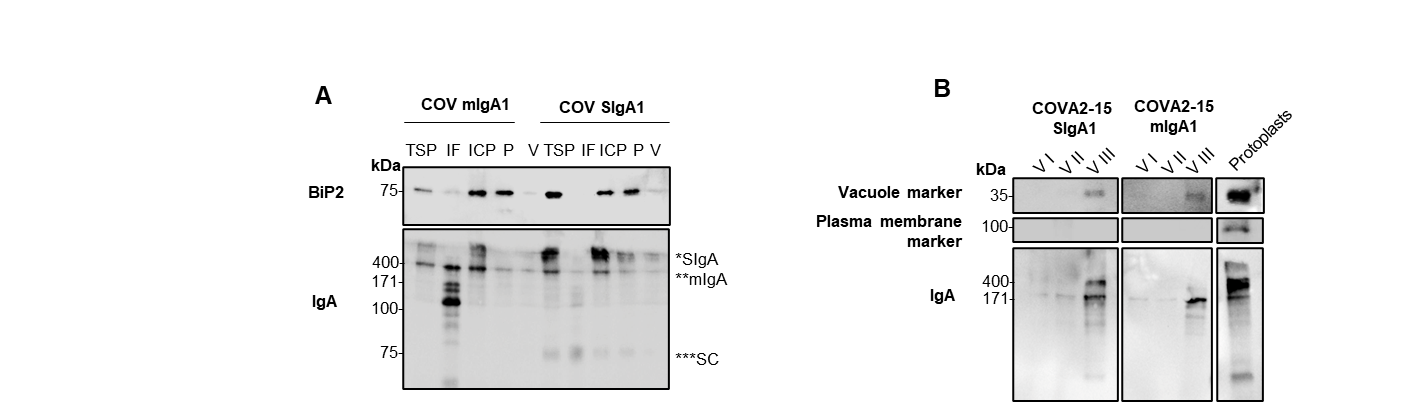


**Figure S5:** Non-reducing SDS-PAGE followed by immunoblotting of vacuolar fractions (VI-III). Vacuoles were isolated from *N. benthamiana* WT leaves infiltrated with monomeric or secretory COVA2-15 IgA1. Protoplasts expressing SIgA were loaded as a control. The IgA1 was detected using an αHC antibody. We also used antibodies to detect the vacuolar marker V-ATPase and the plasma membrane marker H+ATPase. One representative experiment is shown from two biological replicates with similar results.

**Supplementary Tables**

**Table S1:** gRNA target sites.

| **Target gene** | **Accession (NbSC LAB360)** | **Guide ID** | **(Proto)spacer sequence** |
| --- | --- | --- | --- |
| *NbCCT1A* | NbL12g09020.1 | G5 | ACAAGAAAAAGTGGAAGAGA |
| *NbCCT1B* | NbL02g16760.1 |  |  |
| *NbCCT2* | NbL10g07140.1 | G4.2 | TCAAGAGAAAGTGGGAGAAA |

**Table S2**: Primers used for amplification and Sanger sequencing of target gene regions in the ER-engineered plant lines.

|  | |  | |  |
| --- | --- | --- | --- | --- |
| **Name** | **Sequence (5′→3′)** | | **Gene** | |
| 1739-1_F | GGTGATTTAATACCTTGCCTTTCC | | *NbCCT1A* | |
| 1739-1_R | AAGTGCCATTTGAAAACAGACAAC | |  |  |
| 3525.2-1.2_F | CGTGAAGGTATGCTCTTTAGTGTTA | | *NbCCT1B* | |
| 3525-1.2_R | TCACAAACATATACTATGGAGGCTC | |  |  |
| 1559-2_F | AGGAACTTGGTGTTAGCTATGTC | | *NbCCT2* | |
| 1559-2_R | CCAAAGTGAAATCATCCCTTGC | |  |  |

**Table S3**: Primers used to construct the gRNA cassette

| **Name** | **Sequence (5′→3′)** |
| --- | --- |
| SapI-UGR_F | TGCTCTTCCATGTCGAGCTGGCAGACATACT |
| NbCCT-G5_MGR | ACGTCTCACACTTTTTCTTGTCTGCCTATACGGCAGTG |
| NbCCT-G5_MGF | ACGTCTCTAGTGGAAGAGAGTTTCAGAGCTATGCTGG |
| NbCCT-G4.2_MGR | ACGTCTCAACTTTCTCTTGACTGCCTATACGGCAGTG |
| NbCCT-G4.2_MGF | ACGTCTCTAAGTGGGAGAAAGTTTCAGAGCTATGCTGG |
| SapI-UGR_R | TGCTCTTCATGCGATCCACTTGCATAGCGAGTCAG |

**Table S4:** Expression levels of COVA2-15 and 2E8 IgA in WT and ER-engineered lines. The total IgA1 levels and the amount of fully-assembled SIgA1 are shown. Mean values represent two technical replicates of three biological replicates for each line. Statistical significance was determined by one-way ANOVA comparing WT to mt1, mt2 and mt3 groups (ns, not significant, **p < 0.01, ***p < 0.001; ****p < 0.0001). LFW = leaf fresh weight.

|  |  | **WT** | | | **mt1** | | | **mt2** | | | **mt3** | | |
| --- | --- | --- | --- | --- | --- | --- | --- | --- | --- | --- | --- | --- | --- |
|  |  | **mg/kg LFW** | | | | | | | | | | | |
| **COVA2-15** | total IgA | 136.2 | ± | 32.32 | 515.3 | ± | 145.1 | 592.3 | ± | 153.7 | 319.6 | ± | 293.9 |
|  | SIgA | 56.74 | ± | 39.79 | 379.3 | ± | 71.26 | 283.4 | ± | 128.4 | 318.7 | ± | 77.61 |
| **2E8** | total IgA | 267.4 | ± | 155.5 | 259.8 | ± | 212.7 | 250.3 | ± | 145.1 | 220.7 | ± | 136.1 |
|  | SIgA | 40.52 | ± | 15.64 | 174.4 | ± | 59.86 | 158.2 | ± | 79.77 | 128.5 | ± | 32.47 |

**Table S5: Site-specific *N*-glycosylation of COVA2-15 and 2E8 SIgA1 in WT *N. benthamiana* and mt1 lines.** Quantification is a representative of two repeats from two different purification batches with similar results.

|  | NLT | | | | NVS | | | | NIS | | | | NLT+NGT | | | | NDT | | | | NYT | | | | NGT | | | | NVT | | | |
| --- | --- | --- | --- | --- | --- | --- | --- | --- | --- | --- | --- | --- | --- | --- | --- | --- | --- | --- | --- | --- | --- | --- | --- | --- | --- | --- | --- | --- | --- | --- | --- | --- |
|  | COVA2-15 | | 2E8 | | COVA2-15 | | 2E8 | | COVA2-15 | | 2E8 | | COVA2-15 | | 2E8 | | COVA2-15 | | 2E8 | | COVA2-15 | | 2E8 | | COVA2-15 | | 2E8 | | COVA2-15 | | 2E8 | |
|  | WT | mt1 | WT | mt1 | WT | mt1 | WT | mt1 | WT | mt1 | WT | mt1 | WT | mt1 | WT | mt1 | WT | mt1 | WT | mt1 | WT | mt1 | WT | mt1 | WT | mt1 | WT | mt1 | WT | mt1 | WT | mt1 |
| Non-glycosylated |  |  |  |  | 16.7 | 12.0 | 28.1 | 24.8 | 0.6 | 1.0 | 0.7 | 0.5 |  |  |  |  | 0.9 | 0.8 | 2.4 | 1.3 | 5.5 | 7.2 | 15.3 | 13.2 |  |  |  |  |  |  |  |  |
| Single-GlcNAc (Gn) | 0.6 |  |  |  | 2.2 |  | 1.5 | 1.2 | 6.3 | 5.4 | 3.1 | 3.4 |  |  | 0.8 |  |  |  |  |  | 4.7 | 4.4 | 3.1 | 3.0 | 2.4 | 2.4 | 1.8 | 1.8 | 4.6 | 3.3 | 3.5 | 3.7 |
| Man4 | 0.9 | 3.6 |  |  | 1.8 | 1.9 | 1.2 | 1.2 | 5.0 | 5.1 | 4.1 | 2.1 |  |  | 0.5 |  |  |  |  | 1.1 | 0.6 | 0.8 | 0.8 | 0.8 | 0.6 | 0.8 | 0.6 | 0.7 | 1.7 | 1.6 | 1.5 | 1.4 |
| Man5 | 2.4 | 2.1 | 1.3 | 0.0 | 4.5 | 4.5 | 3.4 | 3.5 | 9.2 | 8.6 | 6.0 | 6.3 | 1.7 | 1.6 | 2.5 | 2.3 |  | 1.9 | 1.9 | 1.9 | 1.5 | 1.7 | 0.5 | 1.4 | 1.3 | 1.2 | 1.3 | 1.4 | 0.7 | 0.7 | 0.7 | 0.7 |
| Man6 | 11.1 | 8.8 | 13.6 | 10.3 | 6.8 | 6.6 | 5.7 | 6.0 | 11.0 | 10.2 | 8.0 | 8.4 | 9.6 | 9.2 | 8.0 | 7.9 |  |  |  |  | 2.6 | 2.5 | 2.0 | 2.1 | 3.1 | 2.9 | 3.5 | 3.3 | 3.0 | 3.2 | 2.8 | 3.0 |
| Man7 | 7.0 | 5.2 | 4.8 | 3.5 | 3.2 | 17.5 | 2.1 | 2.1 | 36.0 | 37.8 | 31.7 | 32.6 | 7.2 | 7.9 | 6.7 | 6.7 | 15.4 | 16.6 | 8.3 | 6.8 | 6.0 | 6.1 | 2.2 | 2.4 | 12.6 | 13.2 | 14.8 | 14.2 | 6.1 | 7.2 | 6.3 | 6.1 |
| Man8 | 26.9 | 27.0 | 24.4 | 25.2 | 32.0 | 28.4 | 23.0 | 24.0 | 7.3 | 7.0 | 23.8 | 24.6 | 43.6 | 43.3 | 27.7 | 27.5 | 31.5 | 33.6 | 29.5 | 29.2 | 30.7 | 29.7 | 20.1 | 20.5 | 35.5 | 36.7 | 35.3 | 33.5 | 22.9 | 26.6 | 16.8 | 15.5 |
| Man9 | 37.5 | 44.2 | 43.2 | 49.0 | 25.7 | 21.0 | 25.7 | 27.7 | 15.7 | 15.7 | 15.8 | 17.6 | 24.9 | 27.0 | 29.3 | 32.7 | 2.1 | 2.9 | 10.7 | 12.9 | 14.5 | 15.4 | 19.6 | 23.2 | 29.4 | 30.4 | 25.9 | 27.5 | 13.3 | 15.0 | 17.3 | 19.5 |
| Man9+1xHex |  |  |  |  | 0.1 | 0.1 | 0.4 | 0.4 |  |  |  |  |  |  |  |  |  |  |  |  |  |  |  |  |  |  | 1.0 | 1.0 |  |  | 0.8 | 0.9 |
| Man4G |  |  |  |  | 0.3 | 0.3 | 0.4 | 0.3 | 0.4 | 0.5 | 0.4 | 0.4 |  |  |  |  |  |  |  |  |  |  |  |  |  |  |  |  |  |  |  |  |
| Man5Gn |  |  |  |  | 0.5 | 0.5 | 0.6 | 0.5 | 4.2 | 4.3 | 0.0 |  |  |  | 2.1 | 1.0 |  |  |  |  |  |  |  |  |  |  |  |  |  |  | 0.3 | 0.1 |
| MU |  |  |  |  |  |  |  |  | 1.1 | 1.0 | 0.9 | 1.1 |  |  |  |  |  |  |  |  | 0.4 |  |  |  | 0.2 | 0.2 | 0.2 | 0.2 | 1.7 | 1.4 | 1.4 | 1.5 |
| MM | 1.1 | 0.8 |  |  | 0.7 | 0.7 | 0.6 | 0.6 | 2.5 | 2.5 | 2.0 | 2.1 |  |  | 0.3 |  |  |  |  |  |  | 0.8 | 0.5 | 0.5 | 0.5 | 0.4 | 0.6 | 0.5 |  |  |  |  |
| GnM |  |  |  |  |  |  |  | 0.2 | 0.2 | 0.3 |  | 0.2 |  |  |  |  |  |  |  |  |  |  |  |  |  |  |  |  | 0.3 | 0.3 | 0.2 | 0.1 |
| GnGn |  |  |  |  | 0.1 | 0.1 | 0.1 | 0.1 | 0.2 | 0.2 | 0.1 | 0.1 |  |  |  |  |  |  |  |  |  |  |  |  |  |  |  |  | 0.1 | 0.2 |  |  |
| MMX | 1.8 |  |  |  | 0.3 | 0.4 | 0.3 | 0.3 |  |  |  |  |  |  |  |  |  |  |  |  | 1.1 | 1.6 | 3.0 | 0.8 | 0.7 | 0.5 | 0.6 | 0.6 | 1.2 | 1.1 | 1.0 | 1.0 |
| MUX |  |  |  |  |  |  |  |  |  |  |  |  |  |  |  |  |  |  |  |  | 0.7 |  |  |  |  |  |  |  | 0.5 | 0.3 | 0.5 |  |
| GnMX | 9.2 | 7.5 | 11.1 | 10.6 | 0.5 | 0.6 | 0.5 |  | 0.2 | 0.2 | 0.3 | 0.3 |  |  |  |  |  |  |  |  |  |  |  |  |  |  |  |  | 0.4 | 0.9 | 0.8 | 1.6 |
| GnGnX | 0.5 |  |  |  | 0.1 | 0.1 | 0.1 | 0.1 |  |  | 2.8 |  |  |  |  |  |  |  | 2.9 | 3.6 |  |  |  |  |  |  |  |  | 0.3 | 0.4 |  |  |
| AMX | 0.9 | 0.9 | 1.7 | 1.4 | 0.2 | 0.3 |  | 0.2 |  |  |  | 0.0 |  |  |  |  |  |  |  |  |  |  |  |  |  |  |  |  | 0.1 | 0.1 | 0.3 |  |
| MMF |  |  |  |  |  |  |  | 0.2 |  |  |  |  |  |  |  |  |  |  |  |  |  |  |  |  |  |  |  |  | 1.9 | 1.5 | 1.5 | 1.8 |
| GnMF |  |  |  |  | 0.2 | 0.2 |  | 0.2 |  |  |  | 0.1 |  |  |  |  |  |  |  |  | 0.7 | 0.6 | 0.8 | 0.5 |  |  |  |  | 0.9 | 0.8 | 0.6 | 0.7 |
| GnGnF |  |  |  |  | 0.2 | 0.2 | 0.2 | 0.3 |  |  |  | 0.0 |  |  | 0.8 |  |  |  |  |  | 1.1 | 2.0 | 1.0 | 0.9 |  |  |  |  | 0.7 | 0.9 | 0.4 | 0.5 |
| AMF |  |  |  |  |  |  | 0.1 | 0.1 |  |  | 0.1 |  |  |  | 0.4 | 0.4 |  |  |  |  |  |  |  |  |  |  |  |  |  |  | 0.1 |  |
| MMXF |  |  |  |  |  |  |  |  |  |  |  |  | 4.8 | 3.7 | 5.4 | 6.0 | 24.7 | 19.1 | 16.2 | 17.0 | 18.6 | 14.7 | 16.7 | 17.4 | 12.6 | 10.4 | 11.6 | 13.4 | 20.0 | 15.5 | 19.1 | 21.0 |
| MUXF |  |  |  |  |  |  | 0.2 |  |  |  |  |  |  |  |  |  |  |  |  |  |  |  |  |  |  |  |  |  | 2.0 | 1.5 | 2.2 | 2.1 |
| GnMXF |  |  |  |  | 1.3 | 1.5 | 1.9 | 2.0 |  | 0.1 | 0.2 | 0.3 | 7.3 | 6.1 | 10.0 | 10.0 | 16.2 | 16.1 | 20.2 | 19.4 | 3.8 | 5.0 | 6.4 | 5.8 | 1.0 | 0.9 | 2.7 | 2.1 | 9.7 | 8.8 | 14.5 | 12.5 |
| AMXF |  |  |  |  |  | 0.1 |  |  |  |  |  |  | 0.8 | 1.3 | 2.1 | 2.1 |  |  |  |  |  |  |  |  |  |  |  |  | 0.0 | 0.1 | 0.6 | 0.4 |
| GnGnXF |  |  |  |  | 2.4 | 3.0 | 3.8 | 3.9 |  |  |  |  |  |  | 3.3 | 3.3 | 9.2 | 9.1 | 7.9 | 6.8 | 7.6 | 7.7 | 7.9 | 7.5 |  |  |  |  | 7.9 | 8.7 | 7.1 | 6.0 |
|  |  |  |  |  |  |  |  |  |  |  |  |  |  |  |  |  |  |  |  |  |  |  |  |  |  |  |  |  |  |  |  |  |

**References**

Costantini, L.M., Baloban, M., Markwardt, M.L., Rizzo, M.A., Guo, F., Verkhusha, V.V. and Snapp, E.L. (2015) A palette of fluorescent proteins optimized for diverse cellular environments. *Nat Commun* **6**, 7670.
